# *Gshdz4*-*GmU2AFb*-*GmCML27* Regulatory Pathway Reshapes Root System Architecture and Enhances Alkaline Tolerance in Soybean

**DOI:** 10.3390/plants15142191

**Published:** 2026-07-17

**Authors:** Xiaoyu Wang, Yujing Liu, Mengyu Zhou, Yijia Ruan, Teng Zhang, Xiaohuan Sun, Xinlei Du, Yishan Fu, Jintong Wang, Zaib un Nisa, Junfeng Zhang, Lei Cao

**Affiliations:** 1 College of Horticulture, Northeast Agricultural University, No. 600, Changjiang Road, Xiangfang District, Harbin 150030, China; wxy766696114@163.com (X.W.); yujingliu0000@163.com (Y.L.); zhoumengyu2025@163.com (M.Z.); ryj2504019012@163.com (Y.R.); zhangteng0012@163.com (T.Z.); xaozm@yeah.net (X.S.); duxinlei0101@163.com (X.D.); fys01305611@163.com (Y.F.); wjt1482992725@163.com (J.W.); 2 Institute of Molecular Biology and Biotechnology (IMBB), The University of Lahore, Lahore 54000, Pakistan; zaib.nisa@imbb.uol.edu.pk; 3 School of Geography and Tourism, Harbin University, No. 109, Zhongxing Avenue, Nangang District, Harbin 150076, China; jfzhang@hrbu.edu.cn

**Keywords:** *GmU2AFb*, *GmCML27*, splicing factor, EF-hand, alkaline tolerance, co-overexpression

## Abstract

Alkaline soil limits soybean production. This study elucidates the molecular mechanism by which the *Gshdz4*-*GmU2AFb*-*GmCML27* module regulates soybean alkaline tolerance. Transcriptome analysis, yeast one-hybrid and dual-luciferase assays confirm that the HD-Zip transcription factor Gshdz4 binds to the CAATAA motif in the *GmU2AFb* promoter and activates its transcription. Subcellular localization verifies the nuclear distribution of *GmU2AFb*. Overexpression of *GmU2AFb* improves alkaline tolerance by increasing antioxidant enzyme activities and proline levels, reducing MDA accumulation, facilitating root development, and upregulating alkaline-responsive genes, including *GmSOD1*, while gene knockout impairs stress resistance. Combined Y2H, BiFC and LCI assays validate the nuclear protein interaction between *GmU2AFb* and the calcium-binding protein *GmCML27*, and overexpression of *GmCML27* also enhances antioxidant capacity and root growth in soybean. Co-overexpression of the two genes generates obvious synergistic effects; compared with single-gene overexpression lines, co-transgenic plants possess higher antioxidant levels and elevated transcription of downstream alkaline-tolerant genes, accompanied by alleviated growth inhibition under alkaline stress. In summary, *Gshdz4* transcriptionally activates *GmU2AFb*, and the interaction between *GmU2AFb* and *GmCML27* connects RNA splicing with calcium signaling pathways to synergistically trigger downstream defense responses and promote root development, thereby enhancing soybean alkaline tolerance at multiple layers. This work provides candidate genes for molecular breeding of alkali-resistant soybean.

## 1. Introduction

Alkaline soil conditions exert multifaceted stress on plants through a range of interconnected physicochemical and physiological mechanisms, while at the molecular level they trigger complex regulatory responses. The wild soybean (*Glycine soja*) accession G07256 exhibits the capacity to sustain normal growth in severe saline–alkaline environments, even at pH levels exceeding 8.5 [1]. It harbors a rich repertoire of stress-responsive gene clusters and exhibits outstanding alkaline tolerance, making it a valuable material for discovering alkaline tolerance genes [2]. Soybean is a crucial food and oil crop in China, playing a vital role in national food security. Molecular biology techniques can be employed to enhance soybean resistance to both biotic and abiotic stresses. For instance, seed priming with SME (Stress Memory Encoder) biofertilizer induces somatic heat stress memory in soybean via upregulating heat-responsive genes, optimizing physiological performance and improving thermotolerance [3]. Current research on soybean covers diverse areas; for example, *GmMYB14* positively regulates soybean tolerance to alkaline stress by activating the phenylpropane metabolic pathway to maintain iron homeostasis and scavenge reactive oxygen species [4]. The soybean nodule receptor kinase *GmNARK* is induced by alkaline stress and enhances soybean alkaline tolerance by regulating the ROS signaling pathway [5]. Natural variation in *GmPM30* enhances soybean salt tolerance by strengthening protein interactions of *GmLEA1*-*GmLEC1*, and can be applied to molecular breeding improvement of soybeans in saline–alkali lands [6]. *GmCHYR16* regulates soybean tolerance to bicarbonate stress via ubiquitin-mediated degradation of *GmERF71* [7].

Utilizing genetic resources and regulatory mechanisms associated with saline–alkaline tolerance from wild soybean represents an effective strategy for improving the tolerance of cultivated soybean. For example, studies have shown that overexpression of the *GsJAZ2* gene enhances saline–alkaline tolerance in Arabidopsis [8]; *GsSnRK1* acts synergistically with *GsERF7* to significantly improve the saline–alkaline tolerance of soybean hairy root composite plants [9]; *GsCPI14* from wild soybean interacts with *GsCBRLK* and inhibits cysteine protease activity, positively regulating plant tolerance to alkaline stress [10]; *GsEXPA8* enhances soybean tolerance to sodium bicarbonate stress by modulating root system architecture and the expression of alkaline stress-related genes [11]; *GsSnRK1* phosphorylates *GsSRF2* (at T514), promoting its ubiquitination and degradation to enhance soybean salt tolerance [12]. *GsSKP21* from wild soybean positively confers tolerance to alkaline stress in plants via regulating the expression of ABA signaling pathway-related genes [13]. *GsCYP93D1* from wild soybean positively confers alkaline stress tolerance in plants by reinforcing antioxidant capacity and regulating the ABA signaling pathway [14]. Despite a growing number of studies, the molecular and physiological mechanisms underlying alkaline tolerance in soybean remain largely unresolved.

The HD-Zip protein family comprises plant-specific transcription factors divided into four subclasses, with core functions in regulating growth, development, and abiotic stress responses [15,16]. *Gshdz4*, a member of this family, has been demonstrated to markedly enhance stress tolerance under NaHCO_3_ treatment in *Arabidopsis*, soybean, and lupinus when overexpressed. In previous work, our research group identified *Gshdz4*, *GsNAC019*, and *GsEXPA8* as key alkaline tolerance genes in wild soybean (*Glycine soja*) [11,17,18], and progressively elucidated their hierarchical regulatory mechanisms. Yeast one-hybrid assays demonstrated that *Gshdz4* specifically recognizes and binds the CAATAA/CAATTA cis-element in the *AtNAC019* promoter, thereby activating its expression and enhancing alkaline tolerance in transgenic *Arabidopsis* [18]. Further analysis revealed that the *GsNAC019* promoter in wild soybean also contains potential *Gshdz4* binding sites, suggesting that *Gshdz4* directly activates *GsNAC019* transcription [18]. Based on these findings, a “nucleus–nucleus–membrane” multi-level regulatory model was established in wild soybean: *Gshdz4* in the nucleus upregulates *GsNAC019* expression, and *GsNAC019*, acting as an intermediate hub, recognizes the CGTA conserved motif in the promoter of the downstream functional gene *GsEXPA8*, forming a sequential activation pathway of “*Gshdz4*-*GsNAC019*-*GsEXPA8*” that collectively enhances alkaline tolerance in soybean [19]. By integrating RNA-seq and CUT&Tag-seq analyses, *Gshdz4* was further found to directly regulate multiple stress-related genes such as *GmDEAH5* and *GmATG1c*, broadly participating in hormone signaling pathways including ABA and IAA [19]. Under alkali or cadmium stress, *Gshdz4* establishes a broad-spectrum defense mechanism by regulating targets such as *GmSLX8* and *GmGMFL01*, revealing its core function as a master regulator of multiple stresses [19]. Given the lack of HD-Zip family genes in the lupinus genome, our study introduced *GsHZ4* (*Gshdz4*) from wild soybean into lupinus hairy roots via transcriptome analysis and genetic transformation. Overexpression of *GsHZ4* (*Gshdz4*) significantly enhanced antioxidant enzyme activities (CAT, POD, SOD) under NaHCO_3_ stress, reduced malondialdehyde content, and upregulated key responsive genes such as *LaKIN* and *LaMYB34* [20]. This study confirmed that heterologous expression of *GsHZ4* (*Gshdz4*) effectively improves alkaline tolerance in lupinus, providing a new strategy for crop improvement in saline–alkaline soils.

Integrative analysis of prior RNA-seq and CUT&Tag data suggests that *Gshdz4* positively regulates the splicing factor *GmU2AFb*, facilitating its role in enhancing plant tolerance to alkaline stress. In plants, splicing factors process pre-mRNA to generate mature transcripts, ensuring proper protein synthesis and regulating growth, development, and stress responses [21]. For example, *GsSCL30a* from wild soybean, phosphorylated by *GsSnRK1*, enhances splicing activity and confers improved alkaline stress tolerance [22]. Additionally, the splicing regulator *OsRBP11*, upon activation by bacterial TALEs, promotes alternative splicing of *OsNPR3* to produce a truncated variant that antagonizes the defense function of *OsNPR1*, thereby enhancing disease susceptibility in rice [23]. In *Arabidopsis*, the spliceosome component SKIP enhances osmotic tolerance under salt stress by regulating alternative splicing of *NHX1* and *CBL1* and preventing aberrant transcripts with premature stop codons [24]. Meanwhile, in another study, *SAP18* regulates nuclear splicing of leaf development genes but relocates to the cytoplasm under heat stress to form stress granules, thereby coordinating development and heat tolerance in Arabidopsis [25]. In maize, *ZmHsf23* undergoes alternative splicing to produce two transcripts, *Hsf23L* and *Hsf23S*; *Hsf23S* directly activates sHSPs and TIL1 expression, while *Hsf23L* enhances its transcriptional activation capacity through interaction with *Hsf23S*, collectively improving thermotolerance [26]. The nuclear condensate protein EMB1579 in Arabidopsis interacts with the splicing factors SKIP and RBP47C to regulate stem cell maintenance and cell division in the root meristem at both transcriptional and splicing levels [27]. The plant U2AF65B gene participates in abiotic stress responses through alternative splicing and negatively regulates root elongation and ABA-dependent germination inhibition in *Arabidopsis* [28]. Based on these findings, the splicing factor *GmU2AFb* likely acts as a key downstream target of *Gshdz4*. Considering that *Gshdz4* influences root development and that U2AFb family members are reported to regulate root morphology, it is plausible that *GmU2AFb* also contributes to this regulatory pathway.

Through library screening for the splicing factor *GmU2AFb*, we identified *GmCML27* as one of its interacting proteins. *GmCML27*, a member of the EF-hand family of calcium-binding proteins, functions as a critical calcium signal sensor and transducer in plants. By binding or releasing calcium ions, it perceives intracellular calcium concentration changes and transmits signals to downstream target proteins, thereby regulating plant growth, development, and stress responses. Research has revealed that calcium ions (Ca^2+^) function as ubiquitous second messengers, playing critical roles in plant responses to diverse physiological and environmental stimuli. For instance, 262 calcium signaling-related genes containing EF-hand motifs have been identified in soybean, confirming their widespread involvement in stress responses and the Ca^2+^-binding capacity of certain calmodulin-like (CML) proteins [29]. In maize, *ZmNSA1* undergoes proteasomal degradation upon Ca^2+^ binding, which upregulates plasma membrane H^+^-ATPase and promotes SOS1-mediated Na^+^ efflux, thereby enhancing plant tolerance to saline–alkaline stress [30]. In the liverwort *Marchantia polymorpha*, serine phosphorylation in the N-terminal EF-hand region of RBOHB increases its affinity for calcium ions, synergistically activating the protein in a Ca^2+^-dependent manner—a mechanism conserved in land plants [31]. Additionally, the rice calcium-binding protein *OsCCD1* binds Ca^2+^ and positively regulates seedling tolerance to salt and osmotic stress by modulating the expression of downstream stress-responsive genes [32]. The wild soybean calcium-binding protein *GsCML27*, which contains four EF-hand domains, enhances tolerance to bicarbonate stress when ectopically expressed in *Arabidopsis*, yet reduces tolerance to salt and osmotic stress during seed germination and early growth stages [33]. In this study, we found that *GsCML27* and *GmCML27* share 99.45% sequence similarity, suggesting that *GmCML27* identified in this work is also involved in the regulation of alkali stress responses.

It is speculated that the overexpression of *GmU2AFb* and *GmCML27* can significantly enhance the tolerance of soybean to alkaline stress. Nevertheless, whether the co-overexpression of these two genes can further synergistically improve soybean resistance to alkaline stress remains to be elucidated. Accumulating studies have demonstrated that the co-overexpression and interaction of stress-resistant genes constitute a vital regulatory strategy for plants to adapt to diverse abiotic stresses. For example, the co-overexpression and interaction of *AKR2A* and *AVP1* synergistically enhance plant salt tolerance by regulating sodium compartmentalization and auxin polar transport and promoting lateral root development [34]. The co-overexpression of *ZmWRKY104* and *ZmCCaMK* activates antioxidant defense pathways, thereby positively regulating brassinosteroid-mediated salt tolerance in maize [35]. In tomato, the co-overexpression and interaction of *SlDREBA4* and *SlCAB3* strengthen antioxidant defense and heat stress response pathways to positively regulate thermotolerance and improve high-temperature resistance [36]. In soybean, *GmCBL9* and *GmCIPK6* co-express to form a protein complex that phosphorylates and activates downstream *GmAKT1*, which facilitates potassium influx and maintains Na^+^/K^+^ homeostasis, thereby positively modulating soybean salt tolerance [37].

Based on transcriptome sequencing data from *Gshdz4*-overexpressing soybean under alkali treatment, we observed that *GmU2AFb* was significantly upregulated under alkaline stress, leading us to hypothesize that it might be a key downstream target gene of *Gshdz4*. By overexpressing and knocking out *GmU2AFb* in cultivated soybean roots, we found that *GmU2AFb* overexpression enhances root tolerance to alkaline stress. To further elucidate the functional role of *GmU2AFb*, its interacting protein partners were systematically identified and functionally characterized. Meanwhile, the potential mechanism underlying the coordinated regulation of alkaline tolerance in soybean via co-overexpression of *GmU2AFb* and its interacting proteins was comprehensively investigated.

## 2. Results

### 2.1. Transcriptomic Analysis of GmU2AFb

**Figure 1:**
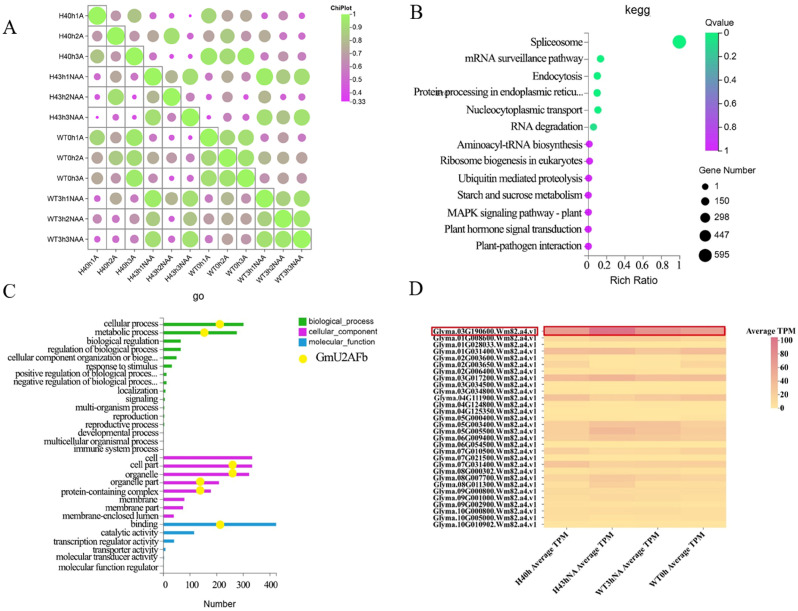
Transcriptomic analysis and functional annotation of *GmU2AFb*. (**A**) Heatmap of correlation analysis among transcriptome samples from soybean plants heterologously overexpressing *Gshdz4*, showing high biological reproducibility between samples. (**B**) Bubble plot of KEGG enrichment analysis of differentially expressed splicing factors, indicating significant enrichment of genes in the spliceosome pathway. (**C**) Bar chart of GO functional classification of *GmU2AFb*, displaying enrichment across the three main categories of biological process, cellular component, and molecular function, with RNA binding identified as the core molecular function. (**D**) Heatmap of expression levels of splicing factor-encoding genes in *Gshdz4*-overexpressing soybean plants, showing high expression level of *GmU2AFb*.

Transcriptomic analysis of soybean plants heterologously overexpressing *Gshdz4* revealed high biological reproducibility and correlation among samples (Figure 1A). A large number of genes encoding splicing factors were identified in the transcript annotation. Functional enrichment analyses using KEGG and GO revealed that the differentially expressed splicing factor genes were significantly enriched in the spliceosome pathway, indicating their primary involvement in mRNA splicing-related biological processes (Figure 1B). Further, Gene Ontology (GO) functional classification indicated that *GmU2AFb* could be categorized into three principal domains: biological process, cellular component, and molecular function. Within the biological process category, *GmU2AFb* was predominantly associated with cellular and metabolic processes; in the cellular component category, it was mainly localized to structures such as cells and organelles; and in the molecular function category, it primarily exhibited binding activity. Among these, RNA binding was identified as the core molecular function of *GmU2AFb*, participating in the recognition of mRNA splice sites (Figure 1C). Transcriptome data further indicated that *GmU2AFb* was highly expressed in the transcriptome (Figure 1D).

### 2.2. GmU2AFb Is a Downstream Target Gene of Gshdz4

**Figure 2:**
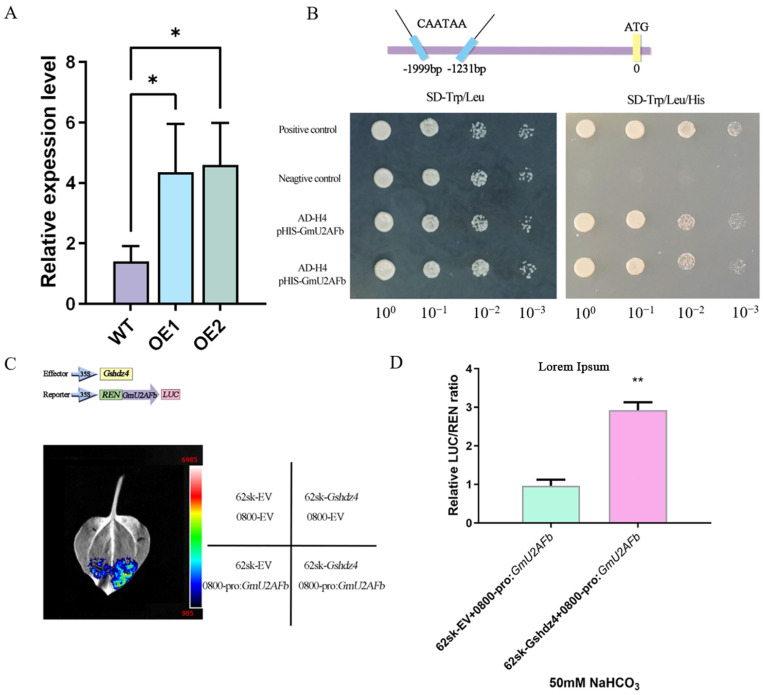
Validation of *GmU2AFb* as a direct downstream target gene of Gshdz4. (**A**) Relative expression levels of *GmU2AFb* in wild-type (WT) and *Gshdz4*-overexpressing (OE) soybean plants after treatment with 50 mM NaHCO_3_ for 6 h, * *p* < 0.05. (**B**) Yeast one-hybrid assay showing yeast growth on SD/-Trp/-Leu and SD/-Trp/-His/-Leu dropout media after co-transformation of the pHIS2 vector containing the *GmU2AFb* promoter fragment with the CAATAA-box and pGADT7-*Gshdz4*. (**C**) Schematic diagram of the luciferase complementation assay, showing the construction of the *62sk*-*Gshdz4* effector vector and the 0800-pro:*GmU2AFb* reporter vector. (**D**) Lucif-erase activity assay after co-overexpression of 62sk-*Gshdz4* and 0800-pro:*GmU2AFb*, with 62sk-EV as the empty vector control, ** *p* < 0.01.

Transcriptomic data from soybean plants heterologously overexpressing *Gshdz4* revealed that *GmU2AFb* expression was significantly upregulated 3 h after alkali treatment, preliminarily confirming that *GmU2AFb*, as a downstream target gene of *Gshdz4*, responds to alkali stress signals (Figure S1). To validate the transcriptomic data, wild-type and *Gshdz4*-overexpressing soybean plants were treated with 50 mM NaHCO_3_ for 6 h. The relative expression level of *GmU2AFb* was significantly higher in the overexpressing plants than in wild-type plants, indicating that *Gshdz4* enhances alkali tolerance in soybean by upregulating *GmU2AFb* (Figure 2A). Yeast one-hybrid assays demonstrated that *Gshdz4* specifically binds to the CAATAA-box within the promoter fragment, as evidenced by the growth of positive yeast colonies on SD/-Trp/-Leu and SD/-Trp/-His/-Leu solid media with the experimental group growing on the triple dropout medium (Figure 2B). Luciferase assays showed that the luminescence value was higher when 62sk-*Gshdz4* was co-expressed with 0800-pro:*GmU2AFb* compared to 62sk-EV co-expressed with 0800-pro:*GmU2AFb*, and the increased luciferase activity indicated that *Gshdz4* expression enhances *GmU2AFb* expression (Figure 2C,D). Both the yeast one-hybrid and luciferase assays confirmed a positive regulatory relationship between *Gshdz4* and *GmU2AFb*. Therefore, it is suggested that *GmU2AFb* is a key downstream target gene of *Gshdz4*.

### 2.3. Protein Structure, Sequence Characteristics, Tissue Expression Pattern, and Subcellular Localization of GmU2AFb

**Figure 3:**
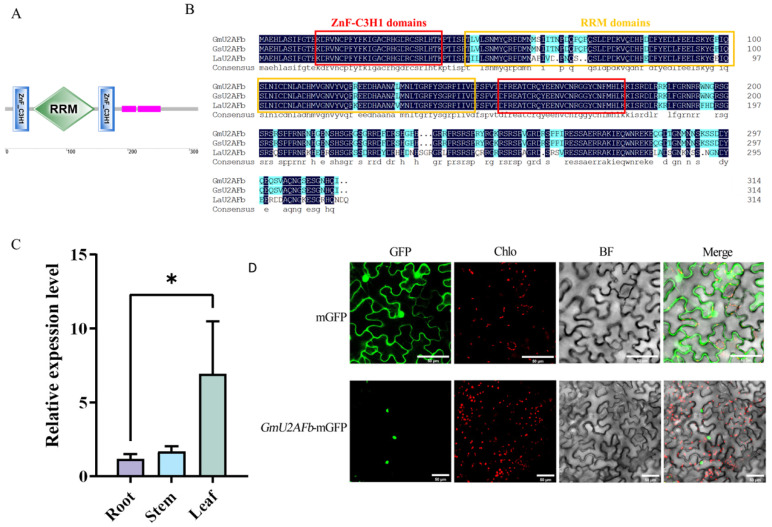
Protein structure, sequence characteristics, tissue expression pattern, and subcellular localization of *GmU2AFb*. (**A**) Schematic diagram of the *GmU2AFb* protein domains, showing that it comprises an N-terminal ZnF-C3H1 domain, a central RRM domain, and a C-terminal ZnF-C3H1 domain. (**B**) Multiple sequence alignment analysis of *GmU2AFb* with its homologs from wild soybean (*GsU2AFb*) and lupinus (*LaU2AFb*). The results showed that the three proteins share 92.19% sequence similarity, with the ZnF-C3H1 domains marked by red boxes and the RRM domain marked by a yellow box. (**C**) Tissue-specific expression analysis of *GmU2AFb* in soybean roots, stems, and leaves. Asterisks indicate significant differences (Student’s *t*-test, * *p* < 0.05). The results show that *GmU2AFb* exhibits the highest expression level in leaves. (**D**) Subcellular localization analysis of *GmU2AFb*. mGFP (control) and *GmU2AFb*-mGFP fusion proteins were expressed in tobacco leaves. Observations were made using GFP fluorescence, chloroplast autofluorescence (Chlo), bright field (BF), and merged images (Merge). Scale bar = 50 μm. The results show that *GmU2AFb* is localized in the nucleus.

Domain analysis of the *GmU2AFb* protein revealed that it consists of an N-terminal ZnF-C3H1 domain, a central RRM (RNA recognition motif) domain, and a C-terminal ZnF-C3H1 domain arranged in tandem, suggesting its potential function in RNA binding and regulation (Figure 3A). Multiple sequence alignment results showed that *GmU2AFb* shares up to 92.19% sequence similarity with its homologs from wild soybean (*GsU2AFb*) and lupinus (*LaU2AFb*). Among these, the ZnF-C3H1 domains (marked by red boxes) and the RRM domain (marked by a yellow box) are highly conserved across different species, further indicating that these domains are critical for the function of *GmU2AFb* (Figure 3B). Tissue expression pattern analysis revealed that *GmU2AFb* is expressed in roots, stems, and leaves of soybean, with expression levels being significantly higher in leaves than in roots and stems (Figure 3C). Subcellular localization experiments further confirmed that the *GmU2AFb*-mGFP fusion protein transiently expressed in tobacco leaves exhibited green fluorescence signals concentrated in the nucleus, whereas the control mGFP was uniformly distributed throughout the entire cell. Combined with observations of chloroplast autofluorescence (Chlo), bright field (BF), and merged images (Merge), with scale bars representing 10 μm, these results clearly demonstrate that *GmU2AFb* is a nuclear-localized protein (Figure 3D).

### 2.4. Overexpression of the Splicing Factor GmU2AFb Enhances Saline–Alkaline Tolerance in Soybean

**Figure 4:**
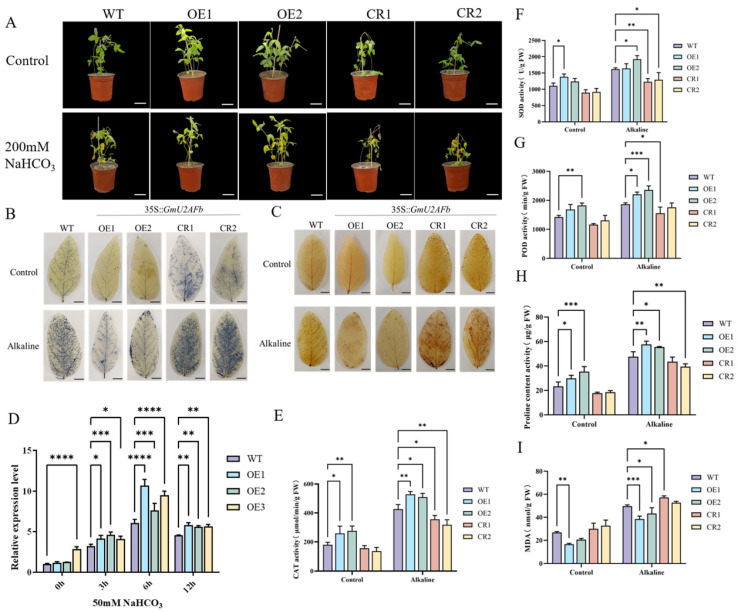
Overexpression of *GmU2AFb* enhances tolerance to alkaline stress in soybean. (**A**) Phenotypic observation of whole plants was performed on wild-type (WT), *GmU2AFb*-overexpressing lines (OE1, OE2) and knockout lines (CR1, CR2) at 15 days after treatment with 0 or 200 mmol·L^−1^ NaHCO_3_. Scale bar = 5 cm. (**B**) NBT staining detecting superoxide anion accumulation in leaves of each line, with staining intensity indicating the degree of oxidative damage. Scale bar = 1 cm. (**C**) DAB staining detecting hydrogen peroxide accumulation in leaves of each line, with staining intensity indicating the degree of oxidative damage. Scale bar = 1 cm. (**D**) Expression levels of *GmU2AFb* in WT and OE plants treated with 50 mM NaHCO_3_ for 0, 3, 6, and 12 h. (**E**–**I**) Alkaline treatment was applied to wild-type (WT), overexpression (OE), and knockout (CR) soybean lines, and the contents of catalase (CAT, (**E**)), superoxide dismutase (SOD, (**F**)), peroxidase (POD, (**G**)), proline (**H**), and malondialdehyde (MDA, (**I**)) were measured. Statistical significance was determined using Student’s *t*-test (* *p* < 0.05, ** *p* < 0.01, *** *p* < 0.001, **** *p* < 0.0001).

To elucidate the functional role of the splicing factor gene *GmU2AFb*, functional analysis was performed. The results showed that compared with wild-type plants, overexpressing plants maintained green leaves and grew more robustly under alkaline stress while knockout plants were significantly shorter than wild-type plants and exhibited faster wilting, chlorosis, and growth arrest under stress treatment (Figure 4A). NBT and DAB staining assays were used to assess leaf damage. Our results revealed that composite hairy root plants overexpressing *GmU2AFb* displayed lower oxidative stress levels in leaves relative to wild-type plants, reflecting more steady and vigorous metabolic status of the whole plant. On the contrary, leaves of *GmU2AFb* knockout hairy root plants showed darker blue-brown staining, indicative of aggravated plant oxidative injury (Figure 4B,C). To further investigate the expression level of *GmU2AFb* under 50 mM NaHCO_3_ treatment at 0, 3, 6, and 12 h, RT-qPCR analysis was performed. The results showed that *GmU2AFb* expression gradually increased within 0–6 h, peaked at 6 h, and then gradually decreased (Figure 4D).

Following alkaline treatment with 200 mmol L^−1^ NaHCO_3_, wild-type (WT), *GmU2AFb*-overexpressing lines (OE1, OE2) and knockout lines (CR1, CR2) displayed significant variations in physiological indices. Under optimal growth conditions, the OE lines exhibited markedly higher activities of SOD, POD and CAT as well as elevated proline concentrations, while their MDA contents were substantially lower relative to WT. After alkaline stress, antioxidant-related parameters were universally increased across all genotypes; the OE lines maintained further enhanced levels of antioxidant enzyme activities and proline accumulation but consistently reduced MDA contents compared with WT. In contrast, the CR lines possessed significantly lower SOD, POD, CAT activities and proline contents alongside remarkably higher MDA levels than WT under both normal and alkaline conditions (Figure 4E–I). Collectively, these results demonstrate that *GmU2AFb* positively improves alkaline tolerance and antioxidant capacity in soybean by boosting antioxidant enzyme activities and proline biosynthesis and restricting excessive accumulation of the lipid peroxidation product MDA, whereas knockout of this gene drastically compromises soybean resistance to alkali-induced oxidative damage.

### 2.5. GmU2AFb Promotes Root System Development in Soybean

**Figure 5:**
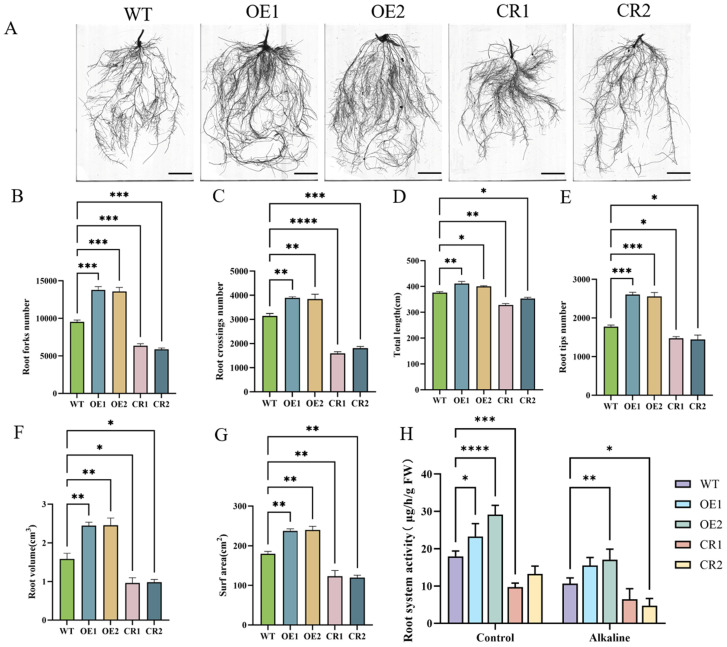
Root scan analysis of wild-type, *GmU2AFb*-overexpressing, and *GmU2AFb*-CRISPR soybean lines. (**A**) Root scan images of wild-type (WT), *GmU2AFb*-overexpressing (OE), and *GmU2AFb*-CRISPR mutant (CR) soybean lines. Scale bar = 5 cm. (**B**) Number of root forks. (**C**) Number of root crossings. (**D**) Total root length. (**E**) Number of root tips. (**F**) Root volume. (**G**) Root surface area. (**H**) Root activity. Error bars represent the standard deviation (SD) of three biological replicates. Statistical significance was determined using Student’s *t*-test (* *p* < 0.05, ** *p* < 0.01, *** *p* < 0.001, **** *p* < 0.0001).

To investigate the effect of *GmU2AFb* on root system development in soybean, root morphology was photographed and quantitatively analyzed using a root scanner. The results showed that overexpression of *GmU2AFb* promoted root system development, resulting in overall larger and denser root architecture, whereas knockout of *GmU2AFb* led to smaller and shorter roots (Figure 5A). Quantitative analysis revealed that compared with wild-type plants, *GmU2AFb*-overexpressing plants exhibited significant increases in root forks, root crossings, total root length, and root tip number. In contrast, all measured root parameters were significantly lower in *GmU2AFb* knockout plants than in wild-type plants (Figure 5B–G). Plants were irrigated with 200 mM NaHCO_3_ solution until distinct phenotypic differences were observed, after which root tissues were collected to determine root activity. Root activity analysis further showed that overexpression of *GmU2AFb* enhanced root activity (Figure 5H). These findings suggest that *GmU2AFb* enhances root growth and development prior to alkali stress, thereby increasing belowground growth potential and providing a foundation for vegetative growth under various environmental stresses.

### 2.6. GmU2AFb Upregulates Alkaline Tolerance-Related Genes in Soybean

**Figure 6:**
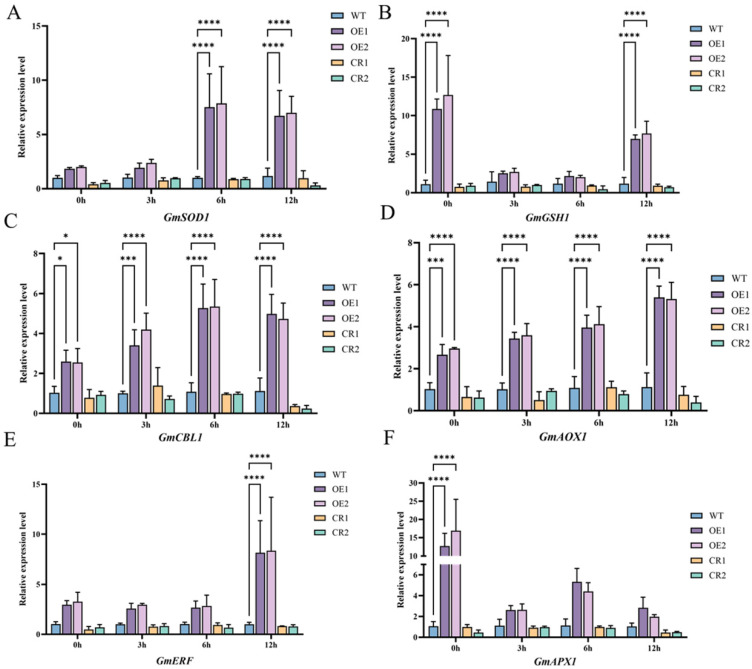
Relative expression levels of alkaline stress-responsive genes in roots of *GmU2AFb* transgenic soybean under normal and alkaline stress conditions. (**A**) *GmSOD1*; (**B**) *GmGSH1*; (**C**) *GmCBL1*; (**D**) *GmAOX1*; (**E**) *GmERF*; (**F**) *GmAPX1*. Error bars represent the standard deviation (SD) of three biological replicates. *GmGAPDH* was used as the reference gene. Statistical significance was determined using Student’s *t*-test (* *p* < 0.05, *** *p* < 0.001, **** *p* < 0.0001).

To investigate the regulatory pathways mediated by *GmU2AFb*, roots of wild-type, overexpression, and knockout lines were treated with 0 or 50 mM NaHCO_3_ solution for 0, 3, 6, and 12 h. The expression levels of key alkaline stress-responsive genes *GmSOD1*, *GmGSH1*, *GmCBL1*, *GmAOX1*, *GmERF*, and *GmAPX1* were determined by RT-qPCR. The expression dynamics of different alkaline-responsive genes exhibit distinct temporal patterns under NaHCO_3_ treatment, which explains the varying significance levels at different time points. The results showed that in the roots of overexpression lines, *GmSOD1* was upregulated at 6 h and 12 h of treatment (Figure 6A), *GmGSH1* was upregulated at 0 h and 12 h (Figure 6B), *GmCBL1* was upregulated at all time points (Figure 6C), *GmAOX1* was upregulated at all time points (Figure 6D), *GmERF* was upregulated at 12 h (Figure 6E), and *GmAPX1* was upregulated only at 0 h (Figure 6F). These results indicate that *GmU2AFb* positively activates a series of alkaline-responsive genes to participate in soybean alkali tolerance. In stark contrast, all marker genes maintained low basal expression in *GmU2AFb*-knockout lines under both normal and alkaline conditions, with no stress-triggered transcriptional elevation or obvious expression fluctuations at any time point. Three distinct regulatory mechanisms may account for this phenomenon. First, *GmU2AFb* functions as a splicing factor at the post-transcriptional level. It amplifies stress signals via alternative splicing rather than regulating basal gene transcription, and knockout of *GmU2AFb* disrupts this signal amplification cascade, leaving marker genes confined to low basal expression. Second, plant post-transcriptional buffering systems only sustain minimal basal transcription and cannot compensate for the loss of stress-inducible gene expression resulting from impaired signal transduction. Third, other independent stress pathways in soybean merely support constitutive basal expression and fail to drive gene upregulation under alkaline stress.

### 2.7. Interaction Between GmU2AFb and GmCML27

**Figure 7:**
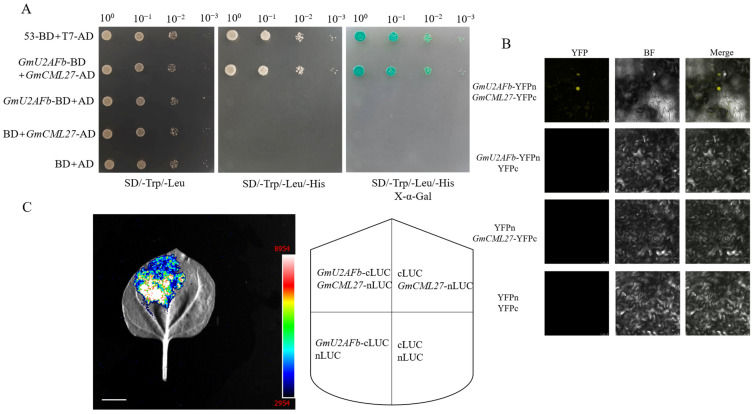
Interaction between GmU2AFb and GmCML27. (**A**) Yeast two-hybrid (Y2H) assay confirming the interaction between GmU2AFb and GmCML27. (**B**) Bimolecular fluorescence complementation (BiFC) assay confirming the interaction between GmU2AFb and GmCML27. YFP: yellow fluorescent protein; BF: bright field; Merge: overlay of YFP and BF. (**C**) Luciferase complementation imaging (LCI) assay. Different combinations were co-infiltrated into distinct areas of tobacco leaves. Only the combination of GmCML27-nLUC and GmU2AFb-cLUC exhibited bright luminescence. Scale bar = 1 cm.

To dissect the molecular mechanism whereby *GmU2AFb* modulates alkaline stress responses, yeast two-hybrid (Y2H) library screening was performed to identify interacting partners of *GmU2AFb*, and preliminary screening indicated a potential physical interaction between *GmU2AFb* and *GmCML27*. Three independent in vivo assays, including Y2H, bimolecular fluorescence complementation (BiFC) and luciferase complementation imaging (LCI), were further employed to verify their interaction. Y2H assays revealed that yeast cells co-transformed with *GmCML27*-AD and *GmU2AFb*-BD grew normally on SD/-Trp/-Leu/-His triple-dropout medium and developed blue colonies upon chromogenic reaction (Figure 7A). BiFC analysis confirmed that their physical interaction occurred in the nucleus of Nicotiana benthamiana mesophyll cells, while robust luminescent signals were captured in LCI assays to validate the protein–protein interaction in plant cells (Figure 7B,C).

### 2.8. Protein Structure, Sequence Characteristics, Tissue Expression Pattern, and Subcellular Localization of GmCML27

**Figure 8:**
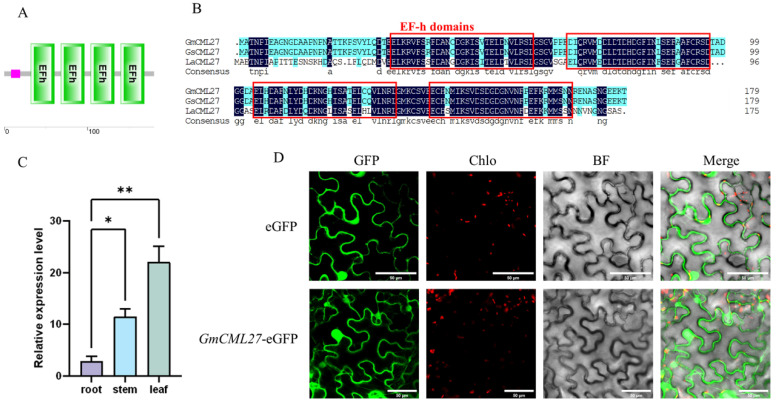
Protein structure, sequence characteristics, tissue expression pattern, and subcellular localization of *GmCML27*. (**A**) Schematic diagram of the *GmCML27* protein domains, showing that it consists of four tandem EF-hand domains. (**B**) Multiple sequence alignment analysis of *GmCML27* with its homologs from wild soybean (*GsCML27*) and lupinus (*LaCML27*). EF-hand domains are marked by red boxes. The results show that the core domains are highly conserved across the three species. (**C**) Tissue-specific expression analysis of *GmCML27* in soybean roots, stems, and leaves. Asterisks indicate significant differences (Student’s *t*-test, * *p* < 0.05, ** *p* < 0.01). The results show that *GmCML27* exhibits the highest expression level in leaves. (**D**) Subcellular localization analysis of *GmCML27*. eGFP (control) and *GmCML27*-eGFP fusion proteins were expressed in tobacco leaves. Observations were made using GFP fluorescence, chloroplast autofluorescence (Chlo), bright field (BF), and merged images (Merge). Scale bar = 50 μm. The results show that *GmCML27* is localized to the plasma membrane, nucleus, and endoplasmic reticulum.

Domain analysis of the *GmCML27* protein revealed that it contains four tandemly arranged EF-hand domains, which typically serve as core calcium ion binding sites, suggesting that *GmCML27* may play a critical regulatory role in calcium ion sensing and signal transduction (Figure 8A). Multiple sequence alignment further showed that *GmCML27* shares high sequence similarity, up to 88.03%, with its homologs from wild soybean (*GsCML27*) and lupinus (*LaCML27*). The EF-hand domains (marked by red boxes) are highly conserved across different species, confirming the evolutionary importance of these calcium-binding domains (Figure 8B). Tissue expression pattern analysis revealed that *GmCML27* is transcriptionally expressed in soybean roots, stems, and leaves, with the highest expression level observed in leaves, followed by stems, and the lowest expression in roots, indicating a distinct tissue-specific expression pattern (Figure 8C). Published studies have verified that *GsCML27* is localized in the nucleus, cell membrane and cytoplasm [33]. Given the sequence identity between *GsCML27* and *GmCML27* reaches up to 99.45%, we initially hypothesized that *GmCML27* shares the same subcellular localization pattern. Subsequent subcellular localization assays further clarified the actual intracellular distribution of *GmCML27* protein. The fusion protein *GmCML27*-enhanced green fluorescent protein (*GmCML27*-eGFP) was transiently expressed in Nicotiana benthamiana leaf epidermal cells. The green fluorescent signals of the fusion protein were visualized and merged with chloroplast autofluorescence (Chlo) and bright field (BF) images. As the negative control, free enhanced green fluorescent protein (eGFP) distributed uniformly throughout the whole cell. Combined with merged images (Merge) with a scale bar of 10 μm, we confirmed that *GmCML27* protein is localized to the cell membrane, nucleus and cytoplasm. (Figure 8D).

### 2.9. GmCML27 Enhances Saline–Alkaline Tolerance in Soybean

**Figure 9:**
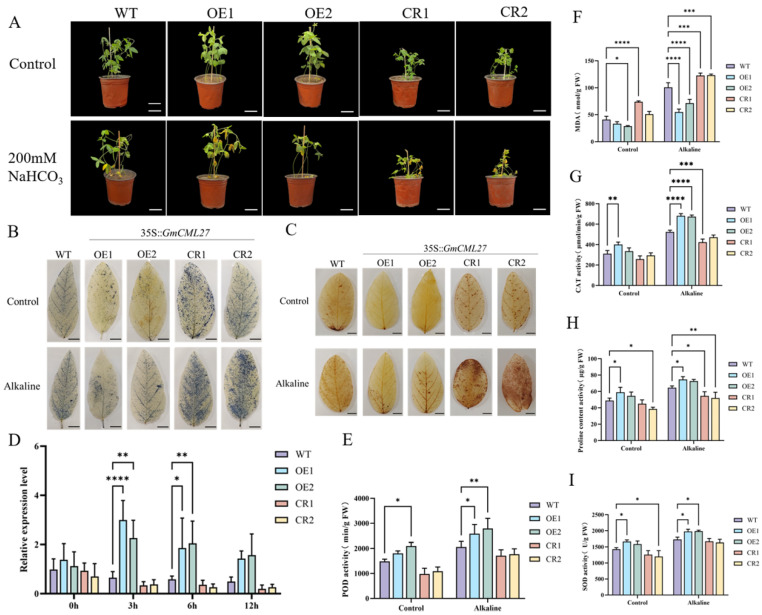
Overexpression of *GmCML27* enhances tolerance to alkaline stress in soybean. (**A**) Phenotypes of whole plants from wild-type (WT), *GmCML27*-overexpressing lines (OE1, OE2) and knockout lines (CR1, CR2) were assessed at 15 days post-treatment with 0 or 200 mmol L^−1^ NaHCO_3_. Scale bar = 5 cm. (**B**) NBT staining detecting superoxide anion accumulation in leaves of each line, with staining intensity indicating the degree of oxidative damage. Scale bar = 1 cm. (**C**) DAB staining detecting hydrogen peroxide accumulation in leaves of each line, with staining intensity indicating the degree of oxidative damage. Scale bar = 1 cm. (**D**) Expression levels of *GmCML27* in WT and OE plants treated with 50 mM NaHCO_3_ for 0, 3, 6, and 12 h. (**E**–**I**) Alkaline treatment was applied to wild-type (WT), overexpression (OE), and knockout (CR) soybean lines, and the contents of peroxidase (POD, (**E**)), malondialdehyde (MDA, (**F**)), catalase (CAT, (**G**)), proline (**H**), and superoxide dismutase (SOD, (**I**)) were measured. Statistical significance was determined using Student’s *t*-test (* *p* < 0.05, ** *p* < 0.01, *** *p* < 0.001, **** *p* < 0.0001).

To investigate the role of *GmCML27* under alkaline stress, *GmCML27* was overexpressed in soybean roots. Phenotypic analysis revealed that under 0 and 200 mM NaHCO_3_ treatment, soybean plants overexpressing *GmCML27* in roots exhibited greener and healthier leaves compared to wild-type plants (Figure 9A). DAB and NBT staining of each line showed that leaves of plants overexpressing *GmCML27* in roots displayed lighter staining, indicating enhanced antioxidant capacity in the leaves (Figure 9B,C). To further examine the expression level of *GmCML27* under 50 mM NaHCO_3_ treatment at 0, 3, 6, and 12 h, RT-qPCR analysis was performed. The results showed that *GmCML27* expression gradually increased within 0–3 h, peaked at 3 h, and then gradually decreased (Figure 9D). Physiological measurements of stress-related parameters showed that under optimal growth conditions, *GmCML27*-overexpressing lines (OE1, OE2) exhibited significantly higher activities of SOD, POD and CAT as well as elevated proline contents, whereas their MDA concentrations were markedly lower relative to wild-type (WT). Following alkaline stress with 200 mmol·L^−1^ NaHCO_3_, antioxidant-related physiological indices were universally upregulated across all genotypes; the OE lines possessed substantially enhanced SOD, POD, CAT activities and proline accumulation but remarkably reduced MDA levels compared with WT. In contrast, knockout lines (CR1, CR2) displayed significantly lower SOD, CAT activities and proline contents than WT under both normal and alkaline treatments. Upon alkaline exposure, CR lines accumulated drastically increased MDA, while no obvious alteration was observed in POD activity (Figure 9E–I). Collectively, these findings verify that *GmCML27* improves the antioxidant capacity of soybean under alkaline conditions, and knockout of this gene compromises plant tolerance to alkaline stress and associated oxidative damage.

### 2.10. GmCML27 Promotes Root System Development in Soybean

**Figure 10:**
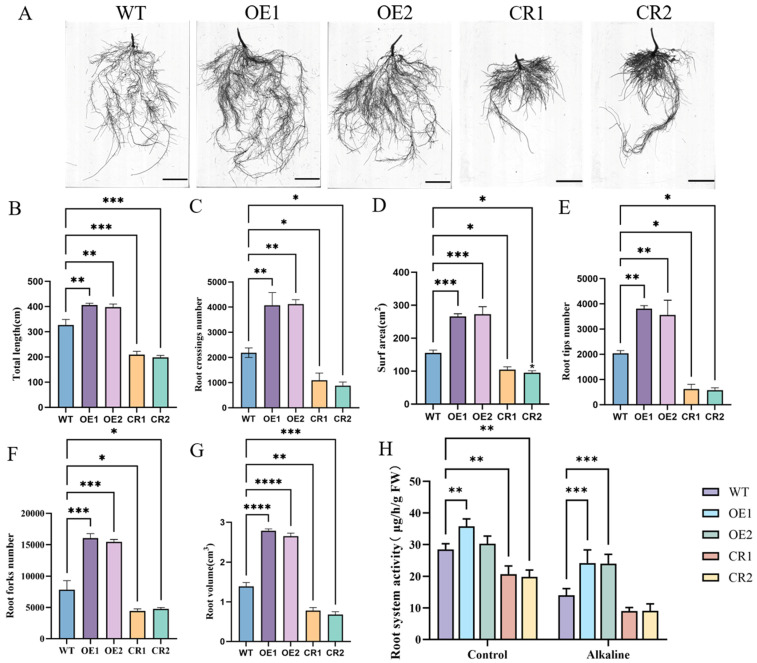
Root scan analysis of wild-type, *GmCML27*-overexpressing, and *GmCML27*-CRISPR soybean lines. (**A**) Root scan images of wild-type (WT), *GmCML27*-overexpressing (OE), and *GmCML27*-CRISPR mutant (CR) soybean lines. Scale bar = 5 cm. (**B**) Total root length. (**C**) Number of root crossings. (**D**) Root surface area. (**E**) Number of root tips. (**F**) Number of root forks. (**G**) Root volume. (**H**) Root activity. Error bars represent the standard deviation (SD) of three biological replicates. Statistical significance was determined using Student’s *t*-test (* *p* < 0.05, ** *p* < 0.01, *** *p* < 0.001, **** *p* < 0.0001).

Given that *GmU2AFb* enhances root development when overexpressed, and given the interaction between *GmU2AFb* and *GmCML27*, we hypothesized that *GmCML27* may possess a similar function. Therefore, roots of *GmCML27*-overexpressing plants were scanned using a root scanner. The results showed that under optimal growth conditions, *GmCML27* overexpression resulted in larger and longer roots, whereas *GmCML27* knockout led to thicker and shorter roots (Figure 10A). Statistical analysis revealed that total root length, number of root forks, root surface area, root tip number, number of root crossings, and root volume were significantly higher in overexpressing plants than in wild-type plants, and significantly lower in knockout plants than in wild-type plants (Figure 10B–G). Root activity assays showed that under optimal growth conditions, *GmCML27*-overexpressing plants exhibited slightly higher root activity, while knockout plants showed significantly reduced root activity; under stress conditions, *GmCML27*-overexpressing plants maintained higher root activity (Figure 10H). These results indicate that *GmCML27* not only enhances plant tolerance to alkaline stress but also improves root activity, thereby contributing to enhanced alkaline stress tolerance.

### 2.11. GmCML27 Modulates the Expression of Genes Associated with Alkaline Stress Tolerance in Soybean

**Figure 11:**
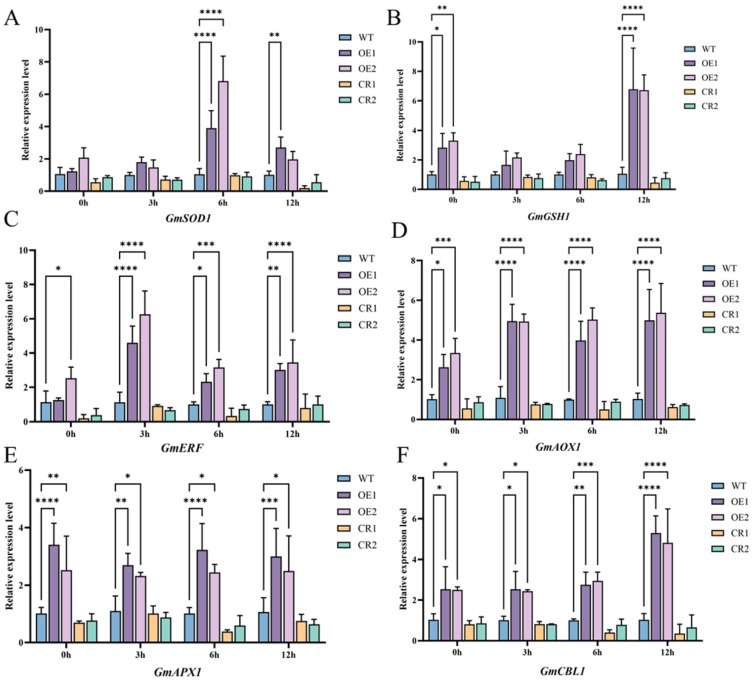
Relative expression levels of alkaline stress-responsive genes in roots of *GmCML27* transgenic soybean under normal and alkaline stress conditions. (**A**) *GmSOD1*; (**B**) *GmGSH1*; (**C**) *GmERF*; (**D**) *GmAOX1*; (**E**) *GmAPX1*; (**F**) *GmCBL1*. Error bars represent the standard deviation (SD) of three biological replicates. *GmGAPDH* was used as the reference gene. Statistical significance was determined using Student’s *t*-test (* *p* < 0.05, ** *p* < 0.01, *** *p* < 0.001, **** *p* < 0.0001).

To investigate the regulatory pathways involving *GmCML27*, we examined the relative expression levels of alkaline tolerance-related genes. The results showed that *GmSOD1* was upregulated at 6 h of alkaline treatment in overexpressing plants (Figure 11A), while *GmGSH1* was upregulated at 0 h and 12 h of treatment (Figure 11B). Expression changes for these two genes were not significant at other time points in overexpressing plants or in knockout plants. For *GmERF* (Figure 11C), *GmAOX1* (Figure 11D), *GmAPX1* (Figure 11E), and *GmCBL1* (Figure 11F), the relative expression levels in overexpressing plants were significantly higher than those in wild-type plants at 0 h, 3 h, 6 h, and 12 h under alkaline treatment, whereas no significant changes were observed in knockout plants compared with wild-type plants. Comparison with the regulatory patterns of *GmU2AFb* revealed that both genes jointly upregulated *GmSOD1* at 6 h, *GmGSH1* at 0 h and 12 h, *GmCBL1* at 6 h and 12 h, *GmAOX1* at 3 h, 6 h, and 12 h, *GmERF* at 12 h, and *GmAPX1* at 0 h under alkaline treatment. Accordingly, it is speculated that the interaction between *GmU2AFb* and *GmCML27* likely functions upstream to coordinately regulate the relative expression levels of the aforementioned genes.

Similar to *GmU2AFb* knockout lines, *GmCML27* knockout lines displayed a consistent phenotype: all marker genes maintained low basal transcript levels, with no stress-induced transcriptional upregulation or obvious expression fluctuations observed at any time point. Several potential factors may contribute to the sustained low expression and lack of stress-triggered fluctuations in *GmCML27* knockout mutants. First, genetic redundancy might partially account for this phenotype. Second, *GmCML27* may amplify alkaline stress signals via calcium perception and physical interaction with *GmU2AFb*, rather than sustaining high basal transcription of target genes. Knockout of *GmCML27* may block the calcium-splicing signaling cascade, and other calcium sensors and stress pathways likely only support extremely low basal transcript abundance. In addition, post-transcriptional compensatory mechanisms in plant cells may merely stabilize basal mRNA levels and fail to restore stress-inducible gene activation. Collectively, the protein complex formed by *GmU2AFb* and *GmCML27* may primarily drive stress-dependent gene upregulation, and this regulatory effect is only prominent under gene overexpression. Disruption of either protein component could interrupt signal amplification, leaving target genes at low basal expression under alkaline stress.

### 2.12. Co-Overexpression of GmU2AFb and GmCML27 Further Enhances Alkaline Tolerance in Soybean

Previous studies have confirmed that individual overexpression of either *GmU2AFb* or *GmCML27* significantly improves alkaline tolerance in soybean, and the two proteins physically interact with each other. Accordingly, we hypothesized that co-overexpression of these two genes could exert synergistic effects and further enhance soybean resistance to alkaline stress. To verify this hypothesis, four types of soybean materials, including wild-type (WT), *GmU2AFb*-single overexpression lines, *GmCML27*-single overexpression lines, and *GmU2AFb*-*GmCML27* co-overexpression lines, were subjected to systematic analyses of plant phenotype, stress-related physiological parameters and transcriptional abundance of alkaline-responsive marker genes under alkaline treatment.

Phenotypic observations revealed that after continuous exposure to 200 mmol·L^−1^ NaHCO_3_ for 20 days, WT plants exhibited severe wilting and partial mortality. Plants overexpressing *GmU2AFb* or *GmCML27* alone survived and kept growing but displayed obvious leaf chlorosis. In contrast, co-overexpression lines presented mildest leaf yellowing, more newly emerging leaves and superior growth status under alkaline conditions, which directly verified the synergistic improvement of alkaline tolerance mediated by dual-gene co-overexpression.

**Figure 12:**
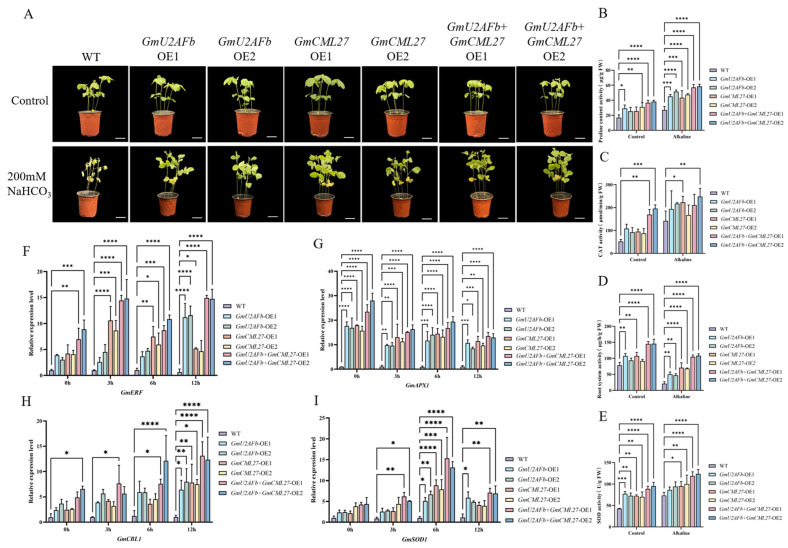
Co-overexpression of *GmU2AFb* and *GmCML27* synergistically enhances alkaline stress tolerance in soybean. (**A**) Whole-plant phenotypes of wild-type (WT), individual *GmU2AFb*-overexpressing lines (OE1, OE2), individual *GmCML27*-overexpressing lines (OE1, OE2), and *GmU2AFb*-*GmCML27* co-overexpressing lines (OE1, OE2) after cultivation under 0 or 200 mmol·L^−1^ NaHCO_3_ for 20 days. Scale bar = 5 cm. (**B**–**E**) Measurements of four stress-associated physiological parameters, including proline content (**B**), CAT activity (**C**), root activity (**D**) and SOD activity (**E**), in various soybean lines under control and alkaline stress conditions. (**F**–**I**) Relative transcript abundances of alkaline-responsive marker genes, namely *GmERF* (**F**), *GmAPX1* (**G**), *GmCBL1* (**H**) and *GmSOD1* (**I**), in different soybean genotypes upon treatment with 50 mmol·L^−1^ NaHCO_3_ for 0, 3, 6 and 12 h, respectively. Statistical significance was determined using Student’s *t*-test (* *p* < 0.05, ** *p* < 0.01, *** *p* < 0.001, **** *p* < 0.0001).

Physiological measurements (Figure 12B–E) indicated that under optimal growth conditions, proline content, CAT activity, root activity and SOD activity were significantly higher in single-gene overexpression lines relative to WT, and these four physiological indices were further elevated in co-overexpression lines. Upon alkaline stress, all genotypes displayed generally increased physiological parameters, among which co-overexpression lines possessed the highest levels of osmotic adjustment and antioxidant-related indicators across all tested lines.

Quantitative real-time PCR results (Figure 12F–I) demonstrated that alkaline stress induced the time-dependent upregulation of four stress marker genes (GmERF, GmAPX1, GmCBL1, GmSOD1). At each sampling time point, the transcript abundances of these marker genes were markedly higher in single-gene overexpression lines than in WT, and co-overexpression lines exhibited significantly elevated gene expression levels compared with both types of single-transgenic lines. Collectively, phenotypic, physiological and molecular evidence confirms that *GmU2AFb* and *GmCML27* function synergistically via protein–protein interaction to positively modulate soybean alkaline tolerance at both physiological and molecular regulatory levels.

## 3. Discussion

### 3.1. Gshdz4 Directly Targets and Regulates GmU2AFb and Its Subcellular Localization

This study confirms that *Gshdz4*, a member of the HD-Zip transcription factor family, directly activates the transcriptional expression of *GmU2AFb* by recognizing and binding to the CAATAA-box element in its promoter region. HD-Zip transcription factors are plant-specific regulators widely involved in growth, development, and stress responses [17]. For example, the rice HD-Zip I transcription factors *Oshox12* and *Oshox14*, orthologs of barley Vrs1, bind to specific DNA sequences and regulate spike development, with overexpression resulting in shortened spikes and reduced plant height [38]; cotton *GhHB12*, induced by auxin, negatively regulates plant height by inhibiting auxin transport and signaling and altering the expression of cell wall extensibility-related genes [39]; *ArHDZ22* in *Anoectochilus roxburghii*, an HD-Zip III transcription factor, negatively regulates plant growth, development, and salt tolerance by downregulating the expression of growth-related genes [40]; members of the HD-Zip I subfamily typically regulate downstream genes by binding to CAAT-motif cis-elements [41], and both soybean *Gshdz4* and Arabidopsis *AtHB1* participate in stress tolerance regulation by recognizing such elements, consistent with the finding in this study that *Gshdz4* binds to the CAATAA-box [17]. Yeast one-hybrid and luciferase assays further validated the direct regulatory relationship, establishing *GmU2AFb* as a direct downstream target gene of *Gshdz4*.

*GmU2AFb* encodes the large subunit of the U2AF splicing factor, which is responsible for recognizing the 3′ splice site during pre-mRNA splicing and belongs to the SR protein-related splicing factor family [42]. The role of splicing factors in plant stress responses has garnered increasing attention. For instance, *Arabidopsis U2AF65A* participates in cold stress responses [43]; alternative splicing variants of *CsWRKY21* in tea plants enhance protein accumulation and regulate abscisic acid content by inhibiting ABA synthesis-related genes, contributing to cold tolerance [44]; the Arabidopsis splicing factors SUA and RSN2 are involved in mediating plant immune defense responses by regulating the proper splicing of SNC4 and CERK1 [45]; rice *OsRBP11* promotes alternative splicing of *OsNPR3* to produce a truncated protein that suppresses the defense function of *OsNPR1*, thereby exacerbating bacterial blight susceptibility, and its mutation can restore disease resistance [23]. The direct regulation of a splicing factor gene by *Gshdz4* suggests that it may broadly influence the splicing patterns of alkaline tolerance-related genes at the post-transcriptional level, thereby amplifying its regulatory effects. Subcellular localization revealed that *Gshdz4* is localized in the nucleus, consistent with its function as a transcription factor and providing a spatial basis for its nuclear regulation of *GmU2AFb* transcription.

In summary, this study reveals an alkaline stress response pathway that operates from transcriptional regulation to RNA processing, expands our understanding of the downstream target genes of HD-Zip transcription factors, and establishes a foundation for elucidating the function of *GmU2AFb* within the alkaline tolerance network.

### 3.2. The Splicing Factor GmU2AFb Enhances Alkaline Tolerance in Soybean by Promoting Root Development and the Antioxidant System

This study confirms that *GmU2AFb* participates in a multi-level regulatory network centered on *Gshdz4* as a downstream target gene. As a splicing factor, the core function of *GmU2AFb* is to participate in pre-mRNA splicing by recognizing the 3′ splice site and regulating alternative splicing events, thereby affecting the production of mature mRNA for stress-responsive genes. The downstream alkaline tolerance-related genes examined in this study (*GmSOD1*, *GmGSH1*, *GmCBL1*, *GmAOX1*, *GmERF*, and *GmAPX1*) belong to distinct functional pathways: *GmSOD1* (superoxide dismutase) and *GmAPX1* (ascorbate peroxidase) are key enzymes in the reactive oxygen species (ROS) scavenging system; *GmGSH1* (glutathione synthetase) is involved in maintaining cellular redox homeostasis; *GmAOX1* (alternative oxidase) is involved in antioxidant protection within the mitochondrial respiratory chain; *GmCBL1*, a core component of calcium signaling pathways, senses and transmits calcium signals; and *GmERF* is an ethylene response factor that regulates plant growth, development, and stress responses. *GmU2AFb* positively upregulates a series of alkaline-responsive genes including *GmSOD1*, *GmGSH1* and *GmCBL1*. However, we have not yet examined the alternative splicing patterns of these genes in the present study, and there is no direct experimental evidence to verify that *GmU2AFb* mediates their transcriptional upregulation by modulating pre-mRNA splicing efficiency or transcript stability. Further experiments focusing on alternative splicing will be carried out in our follow-up research to dissect the precise molecular mechanism by which this splicing factor regulates its downstream target genes.

Both *GmU2AFb* and *GmCML27* are key functional genes involved in the response to alkaline stress in soybean. Each significantly enhances the alkaline tolerance of soybean roots, whereas gene knockout leads to a marked decline in alkaline tolerance, indicating that both act as positive regulators in the soybean alkaline stress response pathway. *GmCML27* belongs to the calmodulin-like protein (CML) family, a group of calcium-binding proteins containing EF-hand domains. Calcium ions (Ca^2+^), acting as second messengers, are involved in plant responses to various environmental stimuli, including both biotic and abiotic stresses [46]. As calcium signal sensors, CML family genes perceive changes in intracellular calcium concentration and transmit stress signals to downstream response elements, playing important roles in plant stress responses. For example, *GsCML27* from wild soybean contains four conserved EF-hand domains, is induced by bicarbonate stress, and enhances tolerance to bicarbonate stress when heterologously expressed in *Arabidopsis* [33]. Additionally, Arabidopsis TCH3 (*CML12*), a calcium-binding protein, is induced by touch and temperature stimuli, is highly expressed in root tip growth regions and vascular tissues, and participates in mechanical stimulation responses and root development regulation [47]. Pepper *CaCIPK7* interacts with *CaCBLs*, is regulated by *CaMYB4*/*88*, and enhances drought tolerance by scavenging ROS and mediating ABA signaling pathways [48]. Soybean *GmCIPK10* interacts with *GmCBL4* and positively regulates salt tolerance by enhancing antioxidant capacity and maintaining ion homeostasis [49]. Alfalfa *MsCML70* significantly enhances salt tolerance in Arabidopsis by regulating ion transport, antioxidant, and stress-related gene expression [50]. *SmCML56* in *Salix matsudana* mediates calcium signaling and positively regulates salt tolerance, with overexpression significantly enhancing salt tolerance [51]. These studies provide important references for the function of *GmCML27* in this study. *GmCML27* likely binds calcium ions, undergoes conformational changes, and interacts with downstream target proteins to regulate the expression of stress-responsive genes. In this study, overexpression of *GmCML27* upregulated multiple alkaline tolerance-related genes, including *GmSOD1*, *GmGSH1*, *GmERF*, *GmAOX1*, *GmCBL1* [52], and *GmAPX1*, suggesting that *GmCML27* may broadly activate downstream defense responses through calcium signaling pathways.

Under alkaline stress, plants suffer oxidative damage due to the massive accumulation of intracellular ROS. In this study, soybean plants overexpressing either *GmU2AFb* or *GmCML27* exhibited significantly increased activities of antioxidant enzymes such as SOD, CAT, and POD in roots, elevated levels of osmotic regulators such as proline, and significantly reduced MDA content. These results indicate that both genes alleviate alkaline stress-induced damage to roots by enhancing the antioxidant system, mitigating oxidative damage, and improving cellular osmotic regulation. This finding is consistent with reports that *GsCML27* enhances bicarbonate tolerance by increasing antioxidant capacity. Furthermore, as a downstream target gene of *Gshdz4*, *GmU2AFb* is directly transcriptionally activated by *Gshdz4*, while *GmCML27* acts as its interacting protein, together forming a “*Gshdz4*-*GmU2AFb*/*GmCML27*” cascade regulatory module that integrates transcriptional regulation with calcium signaling, enabling a multi-level response to alkaline stress in soybean. This provides a new perspective on the molecular regulatory mechanisms underlying plant responses to alkaline stress. Notably, the regulation of alkaline tolerance by both genes is centered on roots, aligning with the role of roots as the primary organ for sensing soil alkaline stress. This suggests that the *GmU2AFb*-*GmCML27* alkaline tolerance module represents a specialized root-specific regulatory system for alkaline stress responses in soybean.

### 3.3. The GmU2AFb and GmCML27 Module Influences Root System Development

The root system is the primary organ responsible for water and mineral nutrient uptake in plants. Under alkaline stress, alterations in soil physicochemical properties severely inhibit root development, thereby affecting nutrient absorption and stress tolerance. This study found that *GmU2AFb* and *GmCML27* are not only involved in alkaline stress responses but also significantly regulate root system growth and development in soybean. Moreover, this regulatory effect is observed under both optimal growth conditions and alkaline stress, indicating that both genes are bifunctional, participating in both growth regulation and stress responses. Root morphological analysis revealed that soybean plants overexpressing either *GmU2AFb* or *GmCML27* exhibited significantly higher total root length, root tip number, root fork number, and root volume compared with wild-type plants, whereas knockout plants displayed shorter, sparser root phenotypes and significantly reduced root activity. These results indicate that both genes positively regulate root initiation and elongation, promote root system architecture establishment, enhance root physiological activity, and ensure normal root function, thereby forming a synergistic mechanism integrating “developmental regulation and stress response.”

From a functional perspective, *GmU2AFb* encodes a splicing factor whose core function is to participate in mRNA splicing. Proper splicing of root development-related genes provides the molecular basis for root system architecture formation. Studies in *Arabidopsis* have shown that the splicing factor RDM16 influences root apical meristem activity and root elongation by regulating the alternative splicing of key transcription factors involved in root stem cell maintenance, such as *PLT1* and *PLT2*, as well as splicing events of cytokinin signaling components (e.g., *ARR1*, *ARR2*, and *ARR11*) [53]. The *rdm16* mutant exhibits reduced root apical meristem cell numbers and shorter roots, and exogenous application of cytokinin or expression of the full-length *ARR1* sequence partially restores root growth [53]. Similarly, the splicing factor PORCUPINE/SmE1 regulates temperature-dependent root development by maintaining auxin homeostasis, and its loss significantly alters root apical meristem structure [54]. Based on these findings, it is plausible that *GmU2AFb* may regulate alternative splicing of root development-related transcription factors (such as *PLT* homologs) or hormone signaling pathway genes (such as auxin/cytokinin response factors) in soybean, ensuring their proper expression and thereby promoting root growth.

As a calcium-binding protein, *GmCML27* belongs to the EF-hand calcium signaling family, and calcium signaling is a crucial pathway regulating root cell division, elongation, and differentiation. As a second messenger, changes in intracellular calcium concentration are sensed and transmitted by calcium-binding proteins, which subsequently regulate the expression of downstream root development-related genes. For instance, *Arabidopsis TCH3* (*CML12*) is highly expressed in the root tip growth region and participates in root development regulation [47]; the calcium-binding protein CMI1 rapidly transmits auxin signals to regulate root growth [55]. Additionally, the phosphorylation regulatory module composed of the calcium-dependent protein kinase CPK and the endoplasmic reticulum calcium pump ECA1 integrates calcium signaling with abscisic acid homeostasis to regulate root growth under osmotic stress [56]. Therefore, *GmCML27* may perceive calcium concentration changes induced by alkaline stress or developmental signals, thereby activating downstream root development-related transcription factors or protein kinases to regulate root growth.

The protein interaction between *GmU2AFb* and *GmCML27* may couple mRNA splicing regulation with calcium signaling to coordinately regulate root development-related pathways. It is hypothesized that this interaction may occur through the following cross-regulatory mechanisms: under alkaline stress, *GmCML27* undergoes conformational changes upon sensing calcium signals, interacts with *GmU2AFb*, and may recruit it to specific pre-mRNAs, thereby enhancing the splicing efficiency of *GmU2AFb* on root development-related genes (such as hormone signaling pathway genes or transcription factor genes); alternatively, *GmU2AFb* may influence the expression of calcium signaling pathway components (such as calcium channel proteins or calcium-binding proteins) through splicing regulation, forming a feedback regulatory loop with *GmCML27*. Such a “calcium signaling–splicing regulation” coupling mechanism would enable more precise and efficient molecular regulation of root development, synergistically enhancing root system architecture establishment and alkaline tolerance in soybean. However, this hypothesis requires further experimental validation.

### 3.4. Expression, Antioxidant Function and Research Limitations of GmU2AFb in Leaves

Quantitative tissue expression analysis revealed that *GmU2AFb* was transcriptionally expressed in soybean roots, stems and leaves, with its transcript abundance significantly higher in leaves than in roots and stems (Figure 3C). Such an expression pattern implies that *GmU2AFb* may perform unique physiological functions in leaves, in addition to its regulatory roles in roots under alkaline stress. Most molecular quantification, root morphology and physiological assays in this study were performed using soybean hairy root transformation materials, where bicarbonate stress was applied to root tissues. Nevertheless, stress signals can be transmitted throughout the whole plant, so we conducted NBT and DAB staining on leaf tissues to evaluate the overall oxidative damage of plants under stress. The staining results demonstrated that leaves of *GmU2AFb*-overexpressing lines accumulated less reactive oxygen species (ROS) after alkaline treatment, whereas knockout lines suffered severe oxidative injury (Figure 4B,C). This directly indicates that *GmU2AFb* participates in leaf antioxidant defense by scavenging ROS and alleviating alkali-induced leaf oxidative damage. The leaf antioxidant phenotype serves as a systemic external manifestation of the regulatory effect of root-expressed *GmU2AFb*. Meanwhile, highly abundant leaf-localized *GmU2AFb* can perceive stress signals transported from underground tissues and independently activate leaf defense responses, forming a coordinated regulatory relationship between roots and leaves.

Restricted by the hairy root transformation system, we cannot completely isolate signaling effects between root and leaf tissues in the present study. Hence, it remains unclear whether *GmU2AFb* possesses leaf-specific regulatory cascades, such as independent regulation of leaf development, photosynthetic metabolism or root-independent leaf stress responses. This research direction is worthy of further exploration, and our research group will carry out in-depth relevant investigations in the follow-up work.

### 3.5. Co-Overexpression of GmU2AFb and GmCML27 Synergistically Modulates Alkaline Tolerance in Soybean

Accumulating evidence has demonstrated that combined overexpression of two physically interacting stress-resistant genes can trigger synergistic effects, conferring much stronger stress tolerance than individual gene overexpression, which serves as a vital strategy for genetic improvement of crops against abiotic stresses. For instance, co-overexpression of interacting *CrWRKY57* and *CrABF3* synergistically activates the transcription of *CrCYCD61*, modulates root development and positively enhances drought tolerance in citrus [57]. Protein interaction between co-expressed *ClWRKY61* and *ClLEA55* improves salt tolerance of watermelon via transcriptional regulation of multiple core salt-responsive genes [58]. By physical interaction, *GhBGH2* binds to the transcriptional activation domain of *GhGLK1* and represses its transcriptional activation on downstream salt-tolerant genes, thereby negatively regulating salt resistance in cotton [59].

Previous assays including Y2H, BiFC and LCI have verified the physical nuclear interaction between *GmU2AFb* and *GmCML27* in the present study. Individual overexpression of either *GmU2AFb* or *GmCML27* improves soybean alkaline tolerance by boosting antioxidant enzyme activities and facilitating root growth. Further dual-gene co-overexpression experiments revealed that after prolonged alkaline stress with 200 mmol·L^−1^ NaHCO_3_, *GmU2AFb*-*GmCML27* co-overexpressing soybean exhibited the mildest wilting and leaf chlorosis, accompanied by more newly formed leaves and superior overall growth performance compared with wild-type and single-gene transgenic lines.

Physiological quantification indicated that under both normal and alkaline growth conditions, co-overexpression lines possessed significantly higher activities of SOD and CAT, stronger root vigor and greater proline accumulation relative to two types of single-overexpression materials, alongside further reduced MDA concentration (an indicator of membrane lipid peroxidation). These findings suggested that dual-gene co-overexpression synergistically strengthens antioxidant capacity and osmotic protection to alleviate alkali-triggered oxidative injury. qRT-PCR results illustrated that the transcript abundances of core alkaline-tolerant genes (*GmERF*, *GmAPX1*, *GmCBL1*, *GmSOD1*) were consistently significantly higher in co-overexpression lines than in single-transgenic counterparts across all sampled time points of alkaline treatment.

Mechanistically, as a splicing factor, *GmU2AFb* mediates pre-mRNA maturation and modulates target gene expression at the post-transcriptional level; as an EF-hand calcium-binding protein, *GmCML27* senses cytosolic calcium signals and transduces stress cues downstream. Their physical interaction enables the crosstalk between post-transcriptional RNA splicing and calcium signal transduction pathways. Such two-layered regulatory cascade synergistically activates downstream stress-defensive genes, achieving functional complementarity in promoting root architecture formation, scavenging reactive oxygen species and maintaining cellular homeostasis, and consequently leading to additive improvement of alkaline tolerance.

Collectively, these findings supplement functional evidence supporting the hierarchical regulatory cascade of *Gshdz4*-*GmU2AFb*-*GmCML27*, and imply that pyramiding *GmU2AFb* and *GmCML27* is a feasible molecular breeding strategy for developing alkali-tolerant soybean cultivars.

### 3.6. Speculation on the Synergistic Alkaline Tolerance Mechanism of GmCML27-GmU2AFb Under the Bidirectional Regulatory Pathway of Calcium Signaling and Alternative

*GmCML27* is a calcium-binding protein, while *GmU2AFb* functions as a splicing factor. Our study verified the physical interaction between these two proteins. Beyond their direct protein–protein interaction, they may possess interrelated cross-regulatory functions, which represents a research direction with great potential.

Accumulated published studies have identified two interlocking regulatory axes linking calcium signaling and splicing machinery in plants. On the one hand, abiotic stress induces a sharp elevation of cytosolic Ca^2+^ concentration; calcium-binding proteins bind Ca^2+^ and undergo conformational changes to form functional complexes with splicing factors, thereby globally orchestrating genome-wide alternative pre-mRNA splicing events. For instance, studies on wild soybean have revealed that stress-triggered calcium signals activate the kinase *GsSnRK1*, which phosphorylates the SR-type splicing factor *GsSCL30a*. This phosphorylation strengthens the capacity of *GsSCL30a* to recognize intronic GAAG cis-elements and interact with the U1-70 spliceosome component, systematically modulating the splicing efficiency of numerous stress-related genes and fully elucidating the molecular route through which Ca^2+^ signals reprogram splicing via kinase-mediated modification of splicing factors [22].

On the other hand, stress signals can first activate splicing factors, which target pre-mRNAs of calcium-binding protein genes to execute alternative splicing and generate functionally distinct protein isoforms, thereby retroactively modulating intracellular calcium signal transduction. For example, Dendrobium officinale *DcaCIPK* generates diurnally differentiated transcripts through alternative splicing: full-length functional transcripts dominate calcium signaling under drought during daytime, whereas truncated transcripts repress kinase activity at night, enabling time-dependent feedback regulation of calcium signals [60]. In Vitis amurensis, *VaCML21* produces four protein isoforms via alternative transcription initiation and intron retention. Isoforms v1 and v2 mediate calcium signals in response to salt and osmotic stresses, while v3 and v4 specifically participate in low-temperature signaling. Different isoforms differentially activate cold-responsive DREB and COR genes, which clearly demonstrates that alternative splicing remodels calcium sensor proteins to precisely decode diverse stress-derived calcium signals [61].

The bidirectional regulatory model, in which Ca^2+^ signals activate splicing programs and splicing factors in turn diversify calcium sensor isoforms, has been validated in legumes, grapes and various other plant species. We therefore hypothesize that the *GmCML27*-*GmU2AFb* module fits into this bidirectional cross-regulatory cascade, providing a theoretical reference for dissecting their multilayered functional crosstalk. Whether *GmCML27* and *GmU2AFb* exert dual cross effects (calcium signal-mediated splicing regulation and splicing-dependent remodeling of calcium sensor isoforms) in soybean requires further experimental verification.

Previous research has reported that the calcium sensor *GsCML27* from wild soybean is rapidly induced in roots under bicarbonate alkaline stress; its protein localizes to the plasma membrane, cytoplasm and nucleus. Upon Ca^2+^ binding, *GsCML27* undergoes conformational rearrangement and positively enhances plant alkaline tolerance by maintaining intracellular Na^+^/K^+^ homeostasis [34]. Sequence alignment shows that *GmCML27* characterized in this study shares 99.45% sequence identity with *GsCML27*, indicating highly conserved functions and analogous positive regulatory roles under alkaline stress. Combined with the two established “calcium-binding protein-splicing factor” bidirectional regulatory axes reported in studies, we propose that *GmCML27* and GmU2AFb coordinate alkaline responses in soybean via a “calcium signal perception-splicing regulation coupling” mechanism. Under NaHCO_3_ stress, cytosolic Ca^2+^ concentration surges. *GmCML27*, which contains four conserved EF-hand motifs, binds Ca^2+^ and changes its conformation, then physically interacts with nucleus-localized *GmU2AFb* to assemble a functional complex. This complex synergistically upregulates a set of core alkaline-tolerant genes including *GmSOD1* and *GmCBL1*, elevates the activities of SOD, CAT and POD as well as proline accumulation, and reduces malondialdehyde (MDA) content to alleviate oxidative damage. Meanwhile, it maintains intracellular Na^+^/K^+^ homeostasis, reinforces antioxidant defense and facilitates root development to jointly improve soybean adaptability to alkaline environments. This regulatory module integrates calcium signal transduction and RNA processing, forming a multi-layered rapid response cascade from stress perception to downstream gene expression regulation.

### 3.7. Multiple Potential Reasons for the Lack of Significant Induction of Antioxidant and Calcium Signaling Marker Genes in Wild-Type Hairy Roots Under Short-Term Alkali Stress

Combining the characteristics of our experimental hairy root system, the temporal pattern of alkali stress responses, multi-layer post-transcriptional regulatory networks, polyploid genomic features of soybean, and the molecular function of *GmU2AFb*, we propose several plausible speculations for the unchanged expression of *GmSOD1*, *GmGSH1*, *GmERF*, *GmAOX1*, *GmAPX1* and *GmCBL1* in wild-type soybean hairy roots after 0–12 h NaHCO_3_ treatment.

First, this observation may partly stem from intrinsic limitations of the composite hairy root system. All plant materials in this study are composite soybean plants with hairy roots induced by *Agrobacterium rhizogenes* K599. As the primary organ sensing alkaline signals, wild-type hairy roots possess weak basal stress tolerance, accompanied by generally low basal transcription of endogenous stress-responsive pathways. Even if alkali stress triggers partial signal transduction cascades, the resulting transcriptional upregulation may be too modest to reach statistical significance within our 0–12 h sampling window.

Second, the 0, 3, 6 and 12 h sampling time points may impose temporal restrictions on transcriptional activation. Although alkali stress presumably initiates stress-sensing cascades, the signal strength may be insufficient to rapidly trigger robust transcription of antioxidant and calcium-signaling marker genes within short-term treatment. In contrast, constitutive high expression of *GmU2AFb* or *GmCML27* in overexpression lines may artificially amplify downstream signal flux, conferring more sensitive and pronounced transcriptional responses under identical stress conditions. The expression peaks of these marker genes in wild-type roots may fall outside our sampling range; their transient, low-magnitude induction may also be indistinguishable from basal transcript levels via statistical analysis. Multiple published time-series studies on soybean alkali stress support this speculation. Time-series transcriptome analyses of wild soybean treated with 50 mM NaHCO_3_ have revealed that alkali stress within 0–12 h mostly only activates genes related to early signal transduction, while large-scale transcriptional activation of antioxidant and calcium-signaling genes such as SOD, APX and CBL may predominantly take place after 24 h of treatment [1]. From a post-transcriptional splicing perspective, Wild soybean endogenous SR splicing factor *GsSCL30a* exhibits low basal abundance. Under alkaline stress, overexpression of this splicing regulatory module significantly elevates the transcript levels of antioxidant genes such as *GmSOD* and *GmAPX*, whereas these genes show only marginal induction in wild-type hairy roots [22]. Experiments using the soybean hairy root system induced by *Agrobacterium rhizogenes* revealed that antioxidant genes maintained low basal expression levels in wild-type hairy roots. Overexpression of *GmNARK* significantly elevated the transcript abundance of antioxidant genes such as *GmSOD1* and *GmAPX1*, and simultaneously increased the activities of SOD, CAT and POD antioxidant enzymes [5].

Third, wild-type soybean harbors intrinsic buffering mechanisms at the post-transcriptional and translational levels. The detected marker genes participate in antioxidant defense, redox homeostasis, calcium signal transduction and transcriptional regulation. To sustain basal physiological homeostasis, these genes are tightly modulated by multiple post-transcriptional controls, including the regulation of mRNA stability, alternative splicing and translational repression, which together form an endogenous physiological buffering system. We speculate that even upon perception of early stress signals, transcriptional outputs may be suppressed at the RNA level within 12 h. Physiological responses such as increased enzyme activity may emerge later or be mediated by post-translational protein modifications. Such weak, transient transcriptional induction is widely documented in abiotic stress research, as these expression shifts are often tissue-specific and easily masked by rapid mRNA turnover.

Fourth, genetic redundancy and functional compensation derived from soybean’s ancient polyploid genome may buffer transcriptional fluctuations of downstream target genes. Massive gene duplication events have occurred during soybean evolution, leading to extensive functional redundancy among homologous gene families. We hypothesize that functional complementation between multiple U2AF and CML paralogs can offset the compromised activity of single isoforms and mitigate transcriptional variation in downstream targets. The unique regulatory capacity of specific isoforms such as *GmU2AFb* and *GmCML27* can only be fully manifested upon forced overexpression, thereby markedly activating downstream marker genes. This hypothesis is consistent with our knockout line results: all tested marker genes maintain low basal expression and barely respond to alkali stress, indicating they cannot be autonomously activated and require a specialized signal-amplification cascade that is only fully triggered in overexpression backgrounds.

Finally, this differential expression pattern can be explained by the intrinsic molecular function of *GmU2AFb* as a splicing factor. Instead of directly recruiting RNA polymerase II to initiate transcription, *GmU2AFb* presumably modulates pre-mRNA splicing efficiency and mature mRNA stability. The elevated transcript abundance of downstream genes in overexpression lines is therefore likely attributed to improved splicing efficiency and enhanced stability of mature transcripts, rather than stimulated transcription initiation. Endogenous splicing factors in wild-type plants only retain basal activity sufficient to sustain constitutive low-level expression of target genes, which cannot support massive, significant accumulation of mature mRNA under short-term alkali stress. Collectively, the above multi-layered speculations reasonably explain the prominent stress-triggered transcriptional responses in overexpression lines and the lack of detectable expression fluctuations of related marker genes in wild-type hairy roots subjected to 0–12 h alkali treatment.

## 4. Materials and Methods

### 4.1. Construction of Hairy Root Transformation Vector and Plant Transformation of Soybean

The coding sequences (CDS) of *GmU2AFb* and *GmCML27* were cloned into the pCAMBIA1302 vector using the NcoI restriction site to generate transgenic soybean plants. The recombinant constructs were transformed into Agrobacterium rhizogenes strain K599 (Weidi, Shanghai, China) for subsequent soybean hairy root transformation assays [62]. The hairy root transformation system was adopted to generate transgenic soybean (cv. Dongnong 50, DN50) roots overexpressing *GmU2AFb* and *GmCML27*.

For CRISPR/Cas9-mediated gene knockout experiments, this study employed the modified binary vector pHK2-Cas9-U6 (Biorun, Wuhan, China), which is specifically designed for gene editing in dicotyledonous plants. Dual-target guide RNAs for *GmU2AFb* and *GmCML27* were designed using an online design tool (https://zlab.squarespace.com/guide-design-resources, accessed on 14 October 2025) and directionally inserted into the Eco31I restriction site of the pHK2-Cas9-U6 vector [63]. The constructed recombinant expression vectors were transformed into *Escherichia coli* DH5α (Weidi, Shanghai, China). Following positive clone selection, recombinant plasmids were extracted and subsequently introduced into *Agrobacterium rhizogenes* K599 competent cells. Genetic transformation was performed using the Agrobacterium-mediated root infection method to ultimately obtain CRISPR-edited plants (CR plants). Identification results of CRISPR-edited plants for *GmU2AFb* and *GmCML27* are shown in Supplementary Figures S2–S5.

### 4.2. Plant Growth Conditions and Experimental Treatments

The cultivated soybean variety Dongnong 50 (DN50, preserved by the College of Horticulture, Northeast Agricultural University) was grown in a growth chamber at the College of Horticulture and Landscape Architecture, Northeast Agricultural University, in a soil:vermiculite mixture (1:2). Growth conditions were set as follows: day/night temperature of 25–30 °C, a 16 h photoperiod, and relative humidity maintained at 60–70%.

Alkali stress treatment experiments were performed using wild-type (WT), gene-overexpressing, co-overexpressing and mutant soybean lines. Soybean roots were treated with water or 50 mM NaHCO_3_ solution for 0, 3, 6, and 12 h. Samples were then collected, immediately frozen in liquid nitrogen, and stored at −80 °C [11].

### 4.3. RNA Extraction and qRT-PCR Analysis

Root tissues were collected at 0, 3, 6, and 12 h after alkali treatment, immediately frozen in liquid nitrogen, and stored at −80 °C. Total RNA was extracted using the Trans Zol Up Kit (ET111-01-V2, TransGen Biotech, Beijing, China), and first-strand cDNA was synthesized using the SPARK script II RT Plus Kit (with gDNA Eraser; AG0304-B; Sparkjade Biotechnology, Jinan, China). Quantitative real-time PCR (qRT-PCR) was performed on a CFX384 Real-Time System (Bio-Rad, Hercules, CA, USA) using SYBR Green Master Mix (AH0105-B; Sparkjade Biotechnology, Jinan, China). Relative expression levels were calculated using the 2^−ΔΔCT^ method [64], with GmGAPDH used as the internal reference control.

### 4.4. Protein Subcellular Localization

The CDSs of *GmU2AFb* and *GmCML27* (without stop codons) were amplified using primers containing homology arms to the pCAMBIA1300-GFP vector (SparkJade Taq PCR Master Mix with dye; Sparkjade Biotechnology, Jinan, China) (Supplemental Table S1). The amplified fragments were inserted into the NcoI-linearized pCAMBIA1300-GFP vector using a homologous recombinase (Vazyme, Nanjing, China) to construct the *GmU2AFb*-GFP and *GmCML27*-GFP fusion vectors. The recombinant plasmids pCAMBIA1300-*GmU2AFb*-GFP and pCAMBIA1300-*GmCML27*-GFP, along with the empty vector pCAMBIA1300, were transformed into Agrobacterium tumefaciens strain GV3101 (pSoup-p19) (Weidi, Shanghai, China). Bacterial suspensions containing the plasmids (OD_600_ = 0.6) were collected by centrifugation and resuspended in a bacterial resuspension buffer containing final concentrations of 10 mM MES, 10 mM MgCl_2_, and 100 μM acetosyringone (AS). After incubation at room temperature for 3 h, the mixtures were infiltrated into different areas of tobacco (*Nicotiana benthamiana*) leaves using a 1 mL needleless syringe, and the infiltration sites were marked. Following overnight incubation in the dark, the infiltrated plants were returned to the greenhouse for 48 h [11]. Leaf tissue sections from the infiltrated areas were prepared and mounted on glass slides. Images were captured using a Leica TCS SP8 confocal laser scanning microscope (Leica, Wetzlar, Germany) [11].

### 4.5. Analysis of Antioxidant and Physiological Indices Under Alkali Stress

Two groups of soil irrigation treatments were performed using a 200 mmol/L sodium bicarbonate solution. One group subjected wild-type soybean lines, *GmU2AFb*-overexpressing lines, *GmCML27*-overexpressing (OE) lines and corresponding mutant (CR) lines to alkaline stress for 10 days. The other group treated wild-type plants, *GmU2AFb*-overexpressing plants, *GmCML27*-overexpressing plants, and dual *GmU2AFb* and *GmCML27* co-overexpressing plants with alkaline stress for 14 days. Root samples of soybean plants cultivated under regular watering conditions and those stressed with 200 mmol/L sodium bicarbonate solution are collected separately for subsequent experimental analysis. Antioxidant enzyme activities, including peroxidase (POD), superoxide dismutase (SOD), and catalase (CAT), were determined to evaluate the oxidative stress response. Physiological parameters, such as malondialdehyde (MDA) content and proline (PRO) accumulation, were measured as indicators of membrane damage and osmotic adjustment, respectively. In addition, root activity was assessed to determine the functional status of the root system. POD activity was quantified using a commercial assay kit (No. BN0051-W96; Bainian Chuangxing Biotechnology, Chongqing, China), whereas SOD, CAT, MDA, PRO, and root activity were measured using corresponding assay kits (Geruisi Biotechnology, Suzhou, China), following the manufacturers’ protocols.

### 4.6. RNA-Seq Analysis

Twenty-five-day-old transgenic *Gshdz4* and wild-type (WT) plants were treated with 50 mM NaHCO_3_ or 0.5 mM CdCl_2_, respectively. Root samples (approximately 500 mg) were collected at 0 and 3 h after treatment, immediately frozen in liquid nitrogen, and stored at −80 °C. Total RNA was extracted, and RNA sequencing was performed on a DNBSEQ platform by BGI (Shenzhen, China). Raw reads were subjected to quality filtering and aligned to the soybean reference genome (Williams82.a2.v1) using HISAT2. Differential gene expression analysis was conducted using DESeq2 (v1.4.5). Differentially expressed splicing factor genes were subjected to Gene Ontology (GO) and KEGG pathway enrichment analysis using clusterProfiler, with terms considered significantly enriched at FDR < 0.05. A heatmap was generated using ChiPlot (https://www.chiplot.online, accessed on 18 October 2025) based on the average expression levels of splicing factors in the transcriptome.

### 4.7. Root System Architecture Analysis Using WinRHIZO

A root analysis system (WinRHIZO Bas STD 4800, Regent, Quebec, Canada) was used for high-throughput, automated multi-parameter measurement of soybean roots. Each seedling was carefully washed and placed in a tray with water covering the roots. The scanned images were analyzed to measure total root length, root volume, root fork and cross counts, root tip number, and surface area [11].

### 4.8. Yeast Two-Hybrid (Y2H) Assay

The pGBKT7 vector was digested with SmaI and PstI, while the pGADT7 vector was digested with EcoRI. The coding sequences (CDS) of *GmU2AFb* and *GmCML27* were directionally inserted into the linearized pGBKT7 and pGADT7 vectors, respectively, to successfully construct the recombinant plasmids pGBKT7-*GmU2AFb* and pGADT7-*GmCML27*. The recombinant plasmids were co-transformed into Y2HGold yeast competent cells (Weidi, Shanghai, China). Positive co-transformed colonies were selected on SD/-Trp/-Leu dropout medium and cultured at 30 °C for 3–5 d. Positive yeast cultures were collected, subjected to 10×, 100×, and 1000× serial dilutions, and spotted onto SD/-Trp/-Leu/-His dropout medium (Takara, Shiga, Japan) for interaction visualization. Co-transformation of pGADT7-T7 and pGBKT7-53 served as a positive control. All plates were incubated at 30 °C for 3–5 d, after which the results were observed and recorded [65].

### 4.9. Yeast One-Hybrid (Y1H) Assay

The pHIS2 vector was digested with EcoRI, and the promoter fragment containing the CAATAA-box recognized by *Gshdz4* was ligated into the pHIS2 vector. The open reading frame of *Gshdz4* was fused with the GAL4 activation domain of the yeast expression vector pGADT7 to generate pGADT7-H4. Positive yeast colonies were spotted onto SD/-Trp/-Leu and SD/-Trp/-His/-Leu solid media, with growth on the triple dropout medium indicating a positive interaction [66].

### 4.10. Bimolecular Fluorescence Complementation (BiFC) Assay

The YFPn and YFPc vectors were linearized by single digestion with BamHI, followed by purification and recovery. Specific primers containing homology arms were used to amplify the coding sequences (CDS) of *GmU2AFb* and *GmCML27*, which were then inserted into the linearized vectors via homologous recombination to generate the recombinant expression constructs YFPn-*GmU2AFb* and YFPc-*GmCML27*, ensuring the fusion of *GmU2AFb* with the N-terminal fragment of YFP and *GmCML27* with the C-terminal fragment of YFP [67]. These recombinant vectors were separately transformed into *Agrobacterium tumefaciens* strain GV3101 (pSoup-p19) competent cells (Weidi, Shanghai, China). Co-infiltration was subsequently performed on the leaves of 4-week-old *N. benthamiana* plants. After infiltration, the plants were incubated in the dark for 2 d, and YFP fluorescence signals were observed and captured using a Leica TCS SP8 Confocal Laser Scanning Microscope (Leica, Germany).

### 4.11. Luciferase Complementation Imaging (LCI) Assay

The nLUC and cLUC vectors were linearized by single digestion with BamHI and subsequently purified (Takara, Shiga, Japan). Specific primers with homology arms were used to amplify the coding sequences (CDS) of *GmU2AFb* and *GmCML27*, which were inserted into the linearized vectors via homologous recombination to generate the recombinant constructs cLUC-*GmU2AFb* and nLUC-*GmCML27*. These were subsequently transformed into *Agrobacterium tumefaciens* strain GV3101 (pSoup-p19) [65]. The following negative controls were included: cLUC + nLUC-*GmCML27*, cLUC-*GmU2AFb* + nLUC, and cLUC + nLUC.

Following standard Agrobacterium-mediated transformation procedures, the prepared Agrobacterium cultures, supplemented with acetosyringone (AS) and MES buffer, were co-infiltrated into the leaves of 4-week-old *N. benthamiana* plants. After infiltration, the plants were incubated in the dark overnight and then cultured under normal conditions for 48 h. The tobacco leaves were uniformly sprayed with 1 mM luciferin potassium salt solution (Puxitong, Beijing, China) and incubated in the dark for 5–10 min. Luminescence signals were captured and analyzed using a fully automated chemiluminescence imaging system (Tanon, Shanghai, China).

### 4.12. Dual-Luciferase Transactivation Assay

The pGreenII 62SK vector was linearized by single digestion with PstI, purified, and the CDS of *Gshdz4* was cloned into the pGreenII 62SK vector. The promoter fragment of *GmU2AFb* containing the CAATAA-box recognized by *Gshdz4* was inserted into the pGreenII 0800-LUC vector. Plasmids were introduced into Agrobacterium strain GV3101 (pSoup-p19). Bacterial cultures (OD_600_ = 0.8) were mixed in a 1:1 ratio (effector:reporter) and infiltrated into the leaves of 4-week-old *N. benthamiana* plants. After 72 h, firefly and Renilla luciferase activities were measured using a dual-luciferase assay kit (Yeasen Biotechnology, 11402ES60). Transactivation activity was calculated as the ratio of LUC to REN [19].

### 4.13. Notes

Two sodium bicarbonate treatment regimens were adopted to meet different experimental requirements in this study. The 50 mM NaHCO_3_ root immersion treatment enables rapid stress stimulation via direct exposure of roots to the solution, which was used for RT-qPCR quantification of target genes using root samples collected at multiple time points. The 200 mM NaHCO_3_ soil irrigation treatment creates mild and long-lasting stress buffered by the growing medium, and was applied for long-term phenotypic observation as well as the measurement of physiological indicators including antioxidant enzyme activities, proline, MDA and root activity.

## 5. Conclusions

**Figure 13:**
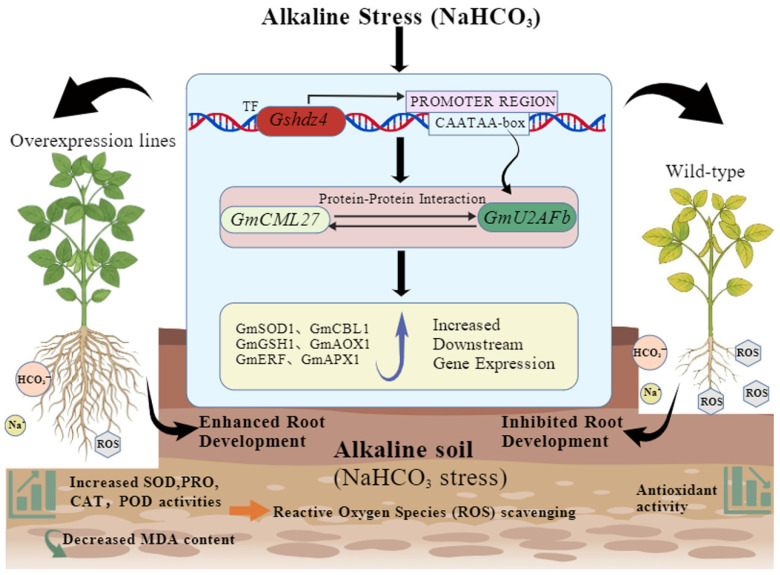
The *Gshdz4*-*GmU2AFb*-*GmCML27* module: a multi-level regulatory model for plant alkaline tolerance. Under alkaline stress (NaHCO_3_), the transcription factor *Gshdz4* is activated and initiates the transcription of its downstream target gene *GmU2AFb* by binding to the CAATAA-box cis-element in the promoter region. *GmU2AFb* interacts with *GmCML27* at the protein level, which upregulates the expression of downstream alkaline tolerance-related genes, including *GmSOD1*, *GmCBL1*, *GmGSH1*, *GmAOX1*, *GmERF*, and *GmAPX1*. High expression of these genes enhances the activities of antioxidant enzymes such as SOD, PRO, CAT, and POD, thereby effectively scavenging reactive oxygen species (ROS) and reducing malondialdehyde (MDA) content. Meanwhile, it significantly promotes root development and improves soybean tolerance to alkaline soil. In contrast, under alkaline stress, wild-type plants lack the above regulatory mechanism, resulting in inhibited antioxidant enzyme activities, excessive ROS accumulation, and impaired root development, ultimately leading to an alkaline-sensitive phenotype. Created with BioGDP.com [68].

As is well known, *Gshdz4*, an HD-Zip transcription factor, directly activates the transcription of *GmU2AFb* by recognizing and binding to the CAATAA-box element in its promoter region. The present study verified that *GmU2AFb*, a nucleus-localized splicing factor, not only responds to alkaline stress but also interacts with the calcium-binding protein *GmCML27* to form a functional module. This module coordinately regulates the expression of a series of alkaline tolerance-related downstream genes, including GmSOD1, GmAPX1, and GmCBL1, significantly increases the activities of antioxidant enzymes (SOD, CAT, POD), reduces oxidative damage (MDA content), and promotes root development (increasing root length, root tip number and root vigor). Moreover, at the physiological level, the *Gshdz4*-*GmU2AFb*-*GmCML27* module enhances stress tolerance and root adaptability in soybean under alkaline stress (Figure 11). Therefore, when plants are subjected to alkaline stress, this module integrates transcriptional regulation, RNA processing and calcium signaling to form a multi-level and highly efficient stress response signaling pathway (Figure 13). Our future work will focus on dissecting how the *GmU2AFb*-*GmCML27* module precisely modulates the splicing and expression of downstream target genes to fully elucidate its potential application value in the genetic improvement of crop stress tolerance.

## Data Availability

The RNA-seq data generated in this study (accession number PRJNA1120591) have been deposited in the Sequence Read Archive (SRA) database of the National Center for Biotechnology Information (NCBI) and made publicly available in our previously published article [19].

## Supplementary Materials

The following supporting information can be downloaded at: https://www.mdpi.com/article/10.3390/plants15142191/s1, Figure S1: the expression level of GmU2AFb in Gshdz4 heterologous overexpression soybean plants under 3 h alkali treatment; Figure S2: Identify overexpression and mutant lines of GmU2AFb via RT-PCR; Figure S3: Sequence chromatogram verification of GmU2AFb mutant lines; Figure S4: Identify overexpression and mutant lines of GmCML27 via RT-PCR; Figure S5: Sequence chromatogram verification of GmCML27 mutant lines; Table S1: the sequences of primers adopted in the article.
