# Supplementary material for: Gshdz4-GmU2AFb-GmCML27 Regulatory Pathway Reshapes Root System Architecture and Enhances Alkaline Tolerance in Soybean
Source: Plants (Basel). 2026 Jul 17;15(14):2191. doi: 10.3390/plants15142191 (PMC13415441; doi:10.3390/plants15142191)
Supplement: Supplementary file 1 [file plants-15-02191-s001.zip › plants-4391998-supplementary.pdf]

Supporting Information

The expression level of *GmU2AFb* in *Gshdz4* heterologous overexpression soybean plants under 3 h alkali treatment is shown in Supplementary Figure S1.

Supplementary Figure S1

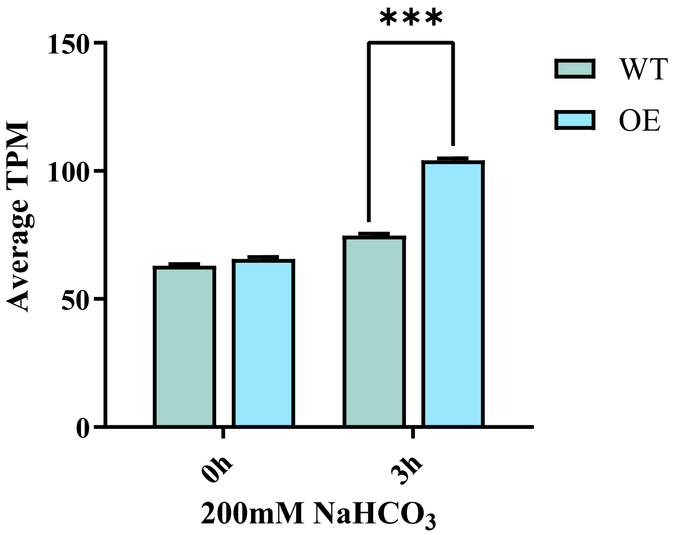

RT-PCR identification showed that bands of *GmU2AFb*-overexpressing plants were brighter in agarose gel electrophoresis, indicating higher transcript abundance, whereas the opposite was observed in CRISPR-edited lines. Sequencing verification revealed that the CRISPR system induced a single-base deletion, which triggered a frameshift mutation in the subsequent coding sequence and ultimately resulted in loss of gene function (Supplementary Figures S2 and S3).

Supplementary Figure S2

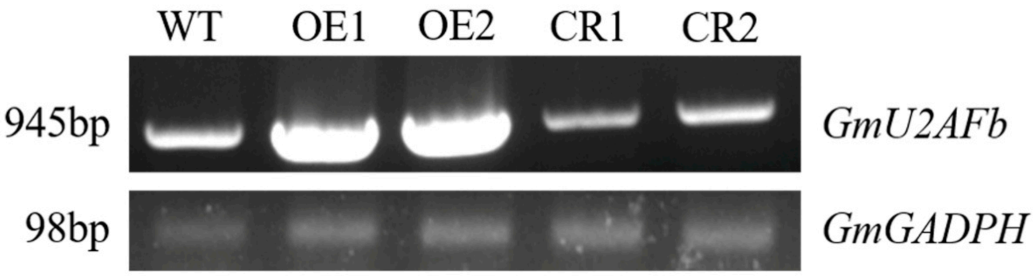

Supplementary Figure S3

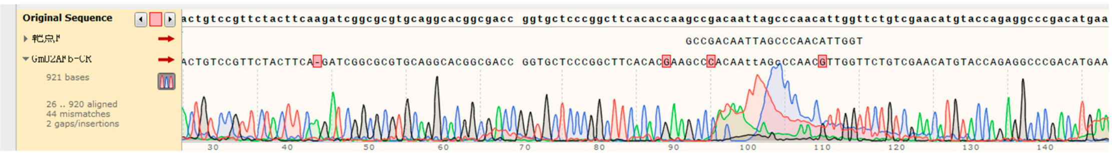

RT-PCR analysis revealed that *GmCML27*-overexpressing plants showed brighter bands during agarose gel electrophoresis, suggesting higher transcript abundance, whereas the CRISPR-edited lines exhibited the opposite result. Sequencing verification confirmed that CRISPR editing caused a 2-base deletion, which led to loss of gene function (Supplementary Figures S4 and S5).

Supplementary Figure S4

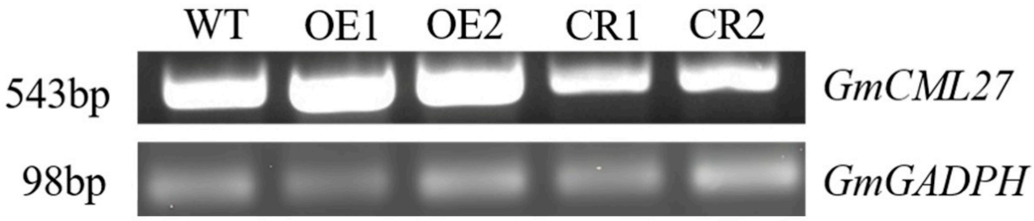

Supplementary Figure S5

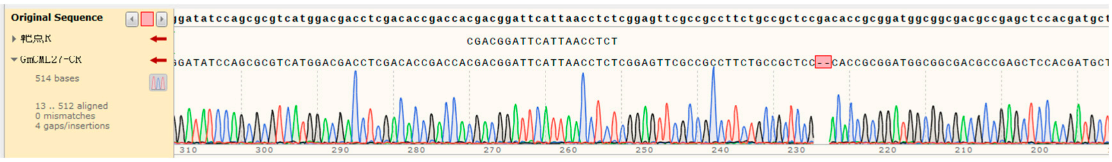

Gene cloning, vector construction, and RT-qPCR primer sequences for *GmU2AFb* and *GmCML27* are listed in Supplementary Table S1:

Primers	Sequence(5’-3’)
GmCML27-1300-F	GAGCTCGGTACCCGGGGATCCATGGCGACGAATCCAATCG
GmCML27-1300-R	CATGTCGACTCTAGAGGATCCATCGGTTTTTTCTTCGCCATT
GmU2AFb-1300-F	GAGCTCGGTACCCGGGGATCCATGGCGGAGCACTTGGCA
GmU2AFb-1300-R	CATGTCGACTCTAGAGGATCCTATCTGATGGTTACCAGATTCACTACC
GmGADPH-F	CCACCGGAGTTTTCACCG
GmGADPH-R	CATGGGGGCATCCTTACTGG
qGmCBL1-F	ATGGGCTGCTACTGCTCAA
qGmCBL1-R	AATGACCCCATTGCGCTTGA
qGmAOX1-F	TGTTCTACCTCCTCTCGCCA
qGmAOX1-R	CATCAGCGCGAATCACAGTG
qGmAPX1-F	AGAAAGGGCTTCTTCGCTCC
qGmAPX1-R	GATTTTGCGAAGTCCTGGCG
qGmGSH1-F	TGAGGAACAGGCCGTACAAC
qGmGSH1-R	AAAAACCCGGTCTGGAAGCC
qGmSOD1-F	AGGAAGGGCTGTTGTTGTCC
qGmSOD1-R	CACAAGCTACTCTGCCACCA
qGmERF-F	CACATCGCTACCCAACAACCC
qGmERF-R	TCTCAACCTGCTCGCTAACTTTG
qGmSnRK1.2-F	CAAGACACAGAACTCCCATTTG
qGmSnRK1.2-R	AAAGGTGAAAAGCAGCCTTAAG
q*GmCML27*-F	CGTCTACCTCCAAGACACGG
q*GmCML27*-R	GTCCATGACGCGCTGGATA
GmCML27-YFPN-F	GAGCTCGGTACCCGGGGATCCATGGCGACGAATCCAATCG
GmCML27-YFPN-R	CATGTCGACTCTAGAGGATCCATCGGTTTTTTCTTCGCCATT
GmCML27-nluc-F	CGAGCTCGGTACCCGGGATCCATGGCGACGAATCCAATCG
GmCML27-nluc-R	GCCGGGCCCTCTAGAGGATCCATCGGTTTTTTCTTCGCCATT
GmU2AFb-YFPC-F	GAGCTCGGTACCCGGGGATCCATGGCGGAGCACTTGGCA

GmU2AFb-YFPC-R	CATGTCGACTCTAGAGGATCCTATCTGATGGTTACCAGATTCACTACC
GmCML27-cris-F	CAGTGGTCTCATGCAACGGCAAGATCTCCGTCACCGTTTTAGAGCTAGAAATAGC
GmCML27-cris-R	CAGTGGTCTCAAAACAGAGGTTAATGAATCCGTCGTGCACCAGCCGGGAATCGAA
GmU2AFb-cluc-F	CCGGGGCGGTACCCGGGATCCATGGCGGAGCACTTGGCA
GmU2AFb-cluc-R	GCCGGGCCCTCTAGAGGATCCCTATATCTGATGGTTACCAGATTCACTACC
GmCML27-AD-F	gccatggaggccagtgaattcATGGCGACGAATCCAATCG
GmCML27-AD-R	atgcccacccgggtggaattcCTAATCGGTTTTTCTTCGCCA
GmCML27-CDS-F	ATGGCGACGAATCCAATCG
GmCML27-CDS-R	CTAATCGGTTTTTCTTCGCCA
GmU2AFb-BD-F	CATGGAGGCCGAATTCCCGGGATGGCGGAGCACTTGGCA
GmU2AFb-BD-R	CTAGTTATGCGGCCGCTGCAGTATCTGATGGTTACCAGATTCACTACC
GmU2AFb-CDS-F	ATGGCGGAGCACTTGG
GmU2AFb-CDS-R	CTATATCTGATGGTTACCAGATTC
GmU2AFb-0800-F	cttgatatcgaattcctgcagGCTATTCTCATTGGAACATGTTGC
GmU2AFb-0800-R	agtggatcccccgggctgcagTCCTTCAGGGTGCCCATTG
GmU2AFb-cris-F	CAGTGGTCTCATGCAGCCGACAATTAGCCCAACATTGGTGTTTTAGAGCTAGAAATAGC
GmU2AFb-cris-R	CAGTGGTCTCAAAACCGTCGAAGTGATCCTGCACCTTGTTGCACCAGCCGGGAATCGAA
GmU2AFb-pHIS-F	attgtaatacgactcactatagggcgGCTATTCTCATTGGAACATGTTGC
GmU2AFb-pHIS-R	ttcgcgaacgcgtgagctccccgggTCCTTCAGGGTGCCCATTG
q*GmU2AFb*-F	AAGGGGAACCCAAACCGAAA
q*GmU2AFb*-R	TTGTCGGGGTCAAGGGATTG
Gshdz4-AD-F	gccatggaggccagtgaattcATGAATCATCGACCACCTTTCC
Gshdz4-AD-R	atgcccacccgggtggaattcCATATACAGATTAATCCATTCCATGCC
Gshdz4-62sk-F	AGTGGATCCCCCGGGCTGCAGATGAATCATCGACCACCTTTCC
Gshdz4-62sk-R	CTTGATATCGAATTCCTGCAGCAGATTAATCCATTCCATGCCG
